# Relative benefit-risk comparing diclofenac to other traditional non-steroidal anti-inflammatory drugs and cyclooxygenase-2 inhibitors in patients with osteoarthritis or rheumatoid arthritis: a network meta-analysis

**DOI:** 10.1186/s13075-015-0554-0

**Published:** 2015-03-19

**Authors:** Anneloes van Walsem, Shaloo Pandhi, Richard M Nixon, Patricia Guyot, Andreas Karabis, R Andrew Moore

**Affiliations:** Mapi, De Molen 84, 3995 AX Houten, The Netherlands; Novartis Pharma AG, Lichtstrasse 35, CH-4002 Basel, Switzerland; University of Oxford, Old Road, Headington, OX3 7LE Oxford, UK

## Abstract

**Introduction:**

There is argument over the benefits and risks of drugs for treating chronic musculoskeletal pain. This study compared the efficacy, safety, and tolerability of diclofenac, ibuprofen, naproxen, celecoxib, and etoricoxib for patients with pain caused by osteoarthritis (OA) or rheumatoid arthritis (RA).

**Methods:**

A systematic literature review used Medline and EMBASE to identify randomised controlled trials. Efficacy outcomes assessed included: pain relief measured by visual analogue scale (VAS); Western Ontario McMaster Universities Arthritis Index (WOMAC) VAS or WOMAC Likert scale; physical functioning measured by WOMAC VAS or Likert scale; and patient global assessment (PGA) of disease severity measured on VAS or 5-point Likert scale. Safety outcomes included: Antiplatelet Trialists’ Collaboration (APTC), major cardiovascular (CV) and major upper gastrointestinal (GI) events, and withdrawals. Data for each outcome were synthesized by a Bayesian network meta-analysis (NMA). For efficacy assessments, labelled doses for OA treatment were used for the base case while labelled doses for RA treatment were also included in the sensitivity analysis. Pooled data across dose ranges were used for safety.

**Results:**

Efficacy, safety, and tolerability data were found for 146,524 patients in 176 studies included in the NMA. Diclofenac (150 mg/day) was likely to be more effective in alleviating pain than celecoxib (200 mg/day), naproxen (1000 mg/day), and ibuprofen (2400 mg/day), and similar to etoricoxib (60 mg/day); a lower dose of diclofenac (100 mg/day) was comparable to all other treatments in alleviating pain. Improved physical function with diclofenac (100 and 150 mg/day) was mostly comparable to all other treatments. PGA with diclofenac (100 and 150 mg/day) was likely to be more effective or comparable to all other treatments. All active treatments were similar for APTC and major CV events. Major upper GI events with diclofenac were lower compared to naproxen and ibuprofen, comparable to celecoxib, and higher than etoricoxib. Risk of withdrawal with diclofenac was lower compared to ibuprofen, similar to celecoxib and naproxen, and higher than etoricoxib.

**Conclusions:**

The benefit-risk profile of diclofenac was comparable to other treatments used for pain relief in OA and RA; benefits and risks vary in individuals and need consideration when making treatment decisions.

**Electronic supplementary material:**

The online version of this article (doi:10.1186/s13075-015-0554-0) contains supplementary material, which is available to authorized users.

## Introduction

Osteoarthritis (OA) and rheumatoid arthritis (RA) are the most common arthritic conditions in adults [1]. Both diseases lead to joint degeneration, are extremely painful, and cause disability and a reduced quality of life [2,3], resulting in a substantial burden to society [4,5].

More than 1.5 billion people worldwide suffer from chronic pain, and arthritic conditions are one of the primary sources for chronic pain. Its prevalence is increasing with an ageing population and pain management is a global public health priority [6,7]. Pain also has multiple serious sequelae, including depression, inability to work, disrupted social relationships, and even suicidal thoughts [7]. Chronic pain and musculoskeletal disorders are associated with some of the poorest health-related quality of life (HRQoL) states ahead of neurological, renal, and cardiovascular (CV) diseases. Patients with pain have a greatly diminished HRQoL, with severe restrictions on their functioning, work, and ordinary activities of daily living [8].

Good pain relief is what patients require from treatment, and this comes with improvement in associated symptoms, function, and quality of life [8,9]. Non-steroidal anti-inflammatory drugs (NSAIDs), both traditional NSAIDs (tNSAIDs) and cyclooxygenase 2 inhibitors (COXIBs) are commonly prescribed to relieve patients from pain and inflammation [2,3]. NSAIDs, both oral and topical, are highly effective analgesics that offer an array of meaningful and differentiated benefits in alleviating pain, and are one of the cornerstones for treating pain in arthritis patients [10,11]. Several pooled analyses and meta-analyses combining randomised trials to estimate the efficacy of an NSAID of interest have been performed [12-16].

In 2004, rofecoxib was withdrawn from the worldwide market due to an increased risk in CV events during chronic use [17]. Since then, the arterial thrombotic risk associated with all NSAIDs, both tNSAIDs and COXIBs, has been subjected to extensive review by medicines regulators, marketing authorization holders, and academic groups around the world [17,18]. Many reviews and (network) meta-analyses have been conducted to investigate safety issues [19-21]. The Coxib and traditional NSAID Trialists’ (CNT) Collaboration has performed meta-analyses on vascular and upper gastrointestinal (GI) effects of NSAIDs. The authors concluded that the vascular risk of high-dose diclofenac, and possibly ibuprofen, are comparable to COXIBs, whereas high-dose naproxen is associated with less vascular risk than other NSAIDs. Additionally, the risk of upper GI complications, especially bleeds, was increased compared to placebo for all COXIBs and tNSAIDs [21].

These meta-analyses have focused mostly on the safety and only a few assessed the efficacy of NSAIDs. None have examined efficacy and safety together. Focusing solely on risks and safety without addressing beneficial effects or investigating only efficacy in the absence of a risk and safety assessment fails to provide a holistic picture of the comparative benefit-risk assessment of NSAIDs. Regulators are also developing and testing tools and processes for balancing multiple benefits and risks as an aid to informed regulatory decisions about benefit-risk assessment of medicinal products [22]. A large number of randomised controlled trials (RCTs) comparing efficacy and safety of NSAIDs to placebo or to each other (head-to-head) exist, and data synthesis methods can be used to combine them into an overall assessment of efficacy and safety.

The objective of this study was to compare the efficacy, safety, and tolerability of commonly used tNSAIDs (diclofenac, ibuprofen, and naproxen) and COXIBs (celecoxib and etoricoxib) in patients with pain caused by OA or RA by means of a Bayesian network meta-analysis (NMA) [23-25]. This study is novel in that a range of different key outcomes were brought together, including efficacy (relief of pain, physical functioning, patient global assessment (PGA)), tolerability (withdrawals), and safety (CV, GI) associated with these treatments in arthritis patients.

## Methods

The Benefit-Risk Action Team, a descriptive framework to conduct benefit-risk assessment, has been followed for structuring and presenting the results of this study [26,27]. The framework provides guidelines on organizing, understanding, and summarizing evidence of benefits and risks into tabular outputs and graphical summaries to allow comparison among treatments.

### Decision context and benefit-risk outcomes identification

The decision context and the scope of the benefit-risk assessment with respect to the population, intervention, comparators, outcomes, and study design (PICOS) were as follows:

- Population: adult patients (≥18 years old) with OA or RA.
- Intervention (Efficacy): diclofenac 75 to 150 mg/day, naproxen 500 to 1,000 mg/day, ibuprofen 1,200 to 2,400 mg/day, celecoxib 100 to 400 mg/day, or etoricoxib 30 to 90 mg/day.
- Intervention (Safety and tolerability): diclofenac 75 to 200 mg/day, naproxen 500 to 1,500 mg/day, ibuprofen 1,200 to 2,400 mg/day, celecoxib 100 to 800 mg/day, or etoricoxib 30 to 90 mg/day.
- Comparators: any of the interventions above compared to each other, placebo, or acetaminophen 4,000 mg/day.
- Efficacy outcomes (Key benefits): pain relief measured by visual analogue scale (VAS), Western Ontario McMaster Universities Arthritis Index (WOMAC) VAS, or WOMAC Likert scale; physical functioning measured by WOMAC VAS or WOMAC Likert scale; PGA of disease severity measured on a VAS or 5-point Likert scale; all outcomes reported at 6 or 12 weeks, within a 2-week range.
- Safety and tolerability outcomes (Key risks): Antiplatelet Trialists’ Collaboration (APTC) events (fatal and non-fatal myocardial infarction (MI) fatal and non-fatal stroke, and other fatal CV events); major CV events (stroke, MI, peripheral arterial thrombosis, peripheral venous thrombosis, pulmonary embolism, and CV-related death); major upper GI events (perforation, obstruction, and gastric and/or duodenal ulcer (includes bleeding ulcers)); withdrawal due to any cause, due to lack of efficacy, or due to adverse events, as reported at the longest follow-up time point.
- Study design: RCTs with study duration ≥2 weeks for efficacy outcomes and ≥4 weeks for safety and tolerability outcomes.

**Figure 1:**
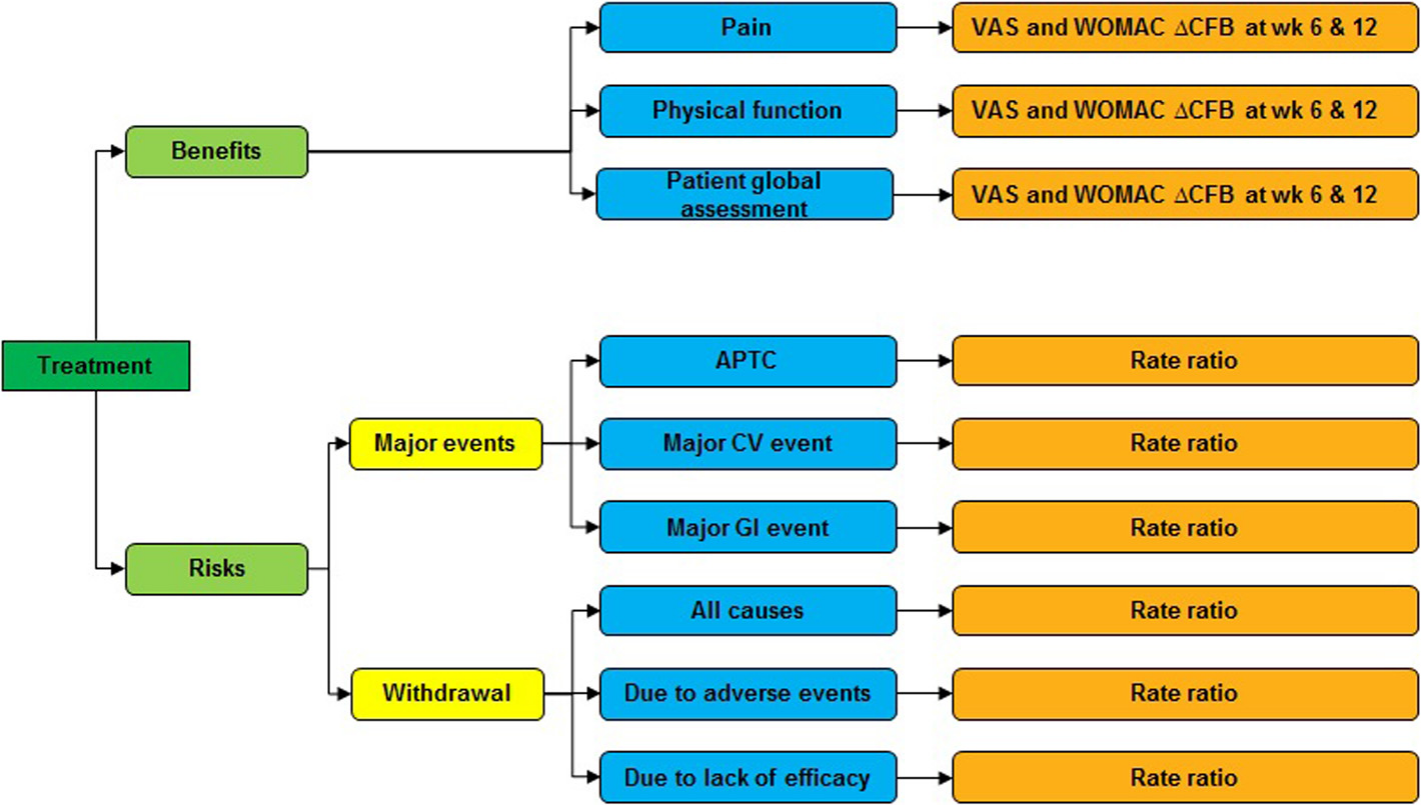
**Benefit-risk value tree.**

A value tree was used to organize the key benefits and risks included in the assessment and drove the benefit-risk balance (Figure 1).

### Identification and collection of source data

The MEDLINE, EMBASE, and Cochrane Library were searched in June 2013 using predefined search strategies (available in Additional file 1). Intervention and study design terms were used, while a search filter was applied to retrieve RCTs [28]. Abstracts and full text articles in a language other than English were excluded.

The relevance of each citation identified was assessed in a two-tiered approach. First, the titles and abstracts were screened for eligibility, and those fulfilling the selection criteria were included in the next stage. The full texts of the selected articles were retrieved and assessed. Those that met the inclusion criteria were included for data extraction. The assessment of each citation was performed by one researcher (AvW) and checked against the original study by another (PG). Any disagreement was resolved by consensus or a third reviewer (AK).

Study and patient characteristics as well as efficacy, safety, and tolerability outcomes from the selected studies were collected in a predesigned data extraction form. Details on study characteristics were extracted, including design; selection criteria; compared interventions; trial duration; number of randomised and intention-to-treat (ITT) patients; and allowance of gastro-protective agents, aspirin, and rescue medication use. Additionally, baseline patient characteristics were extracted, including age, gender, disease duration, history of hypertension and GI ulcer, and percentage of smokers.

For each continuous outcome of interest, the change from baseline (CFB) and the associated sampling variance were extracted. If not available, CFB and standard error were calculated based on the available data (Additional file 2). If necessary, standardized mean differences were calculated as the difference in CFB (ΔCFB) between two interventions divided by the corresponding standard deviation [29]. For dichotomous outcomes, the number of patients experiencing an event was extracted or estimated based on reported percentages and ITT population. Subsequently, the total patient years (pyrs) of follow-up were calculated. Data presented in graphs were extracted using DigitizeIT version 1.5 software (DigitizeIT, Braunschweig, Germany).

The methodological and reporting quality of the included trials was assessed with the Oxford quality scoring system for RCTs [30]. The risk of bias was assessed on the following aspects: randomization according to an appropriate method, allocation concealment of patients and investigators, and complete and non-selective reporting of study withdrawals and dropouts.

### Data synthesis

The selected benefit and risk outcomes, that is, relative efficacy, safety, and tolerability of the treatments of interest were evaluated using a Bayesian NMA [23-25]. Analyses within the Bayesian framework involve data, a likelihood distribution, a model with parameters, and prior distributions for these parameters. In this analysis, a linear model with normal likelihood distribution was used for continuous outcomes and a Poisson likelihood with a log link was used for the dichotomous outcomes [31,32]. Flat (non-informative) prior distributions were assumed for all outcomes. Prior distributions of the relative treatment effects were normal, with zero mean and variance of 10,000, while a uniform distribution with range zero to five was used as the prior of the between-study standard deviation.

It can be expected that there is always some variation in patient characteristics, study sites, and settings across studies. If these characteristics are effect modifiers of the relative treatment effects of interest, there will be heterogeneity in the evidence base [25,33,34]. To allow for heterogeneity between studies, random effects models were evaluated. Random effects models assume that treatment effects may vary between studies, but come from a common distribution of treatment effects, with a mean for each treatment effect and a common between-study covariance matrix.

Furthermore, to address potential bias in our study, a number of scenario analyses were defined *a priori*, while for safety and tolerability only outcomes with at least 50 events reported in total per intervention (across all studies in the network) were analysed. This was done because a low number of events limits the ability of a meta-analysis to detect differences between treatments and can eventually give misleading results [35,36].

For each outcome, fixed and random effects models were evaluated, and the better fitting model was selected based on the lower Deviance Information Criterion value [31]. The posterior densities for unknown parameters were estimated using Markov chain Monte Carlo (MCMC) simulations. All results were based on 80,000 iterations on three chains, with a burn-in of 20,000 iterations. Convergence was assessed by visual inspection of trace plots. The accuracy of the posterior estimates was assessed using the Monte Carlo error for each parameter (Monte Carlo error <1% of the posterior standard deviation). All models were implemented using the WinBUGS version 1.4.3 (MRC Biostatistics Unit, Cambridge, UK) and were based on those defined by Dias *et al*. [32].

The Bayesian NMA provided posterior distributions of the relative treatment effects between interventions and the probability that one treatment is better than another for each outcome of interest. This probability is calculated based on the proportion of MCMC cycles in which the specific treatment estimate is better than the comparator [32]. The results of the NMA are presented in terms of ‘point estimates’ (median of posterior) for the relative treatment effects, along with the 95% credible intervals (95% CrI).

The efficacy outcomes are presented as ∆CFB, with negative values indicating symptomatic improvement of diclofenac relative to comparator. Safety and tolerability results are presented as rate ratios (RR), with RR <1 indicating that diclofenac is associated with a lower risk relative to comparator.

Based on the relative treatment effects resulting from the NMA, a treatment was considered as ‘more effective’ if the point estimate suggested the treatment is expected to be better than the comparator and the 95% CrI does not include 0 (for continuous outcomes) or 1 (for binary outcomes); ‘likely to be favourable’ if the 95% CrI includes 0 or 1 but the point estimate is favourable and there is a ≥85% probability that treatment is better than the comparator; ‘comparable’ if the 95% CrI includes 0 or 1 (probability treatment is better than comparator >15% and <85%); ‘likely to be unfavourable’ if the 95% CrI includes 0 or 1 but the point estimate is unfavourable and there is a ≤15% probability that treatment is better than the comparator; ‘less effective’ if the point estimate suggests the treatment is expected to be worse than the comparator and the 95% CrI does not include 0 or 1 [37].

## Results

### Literature search

**Figure 2:**
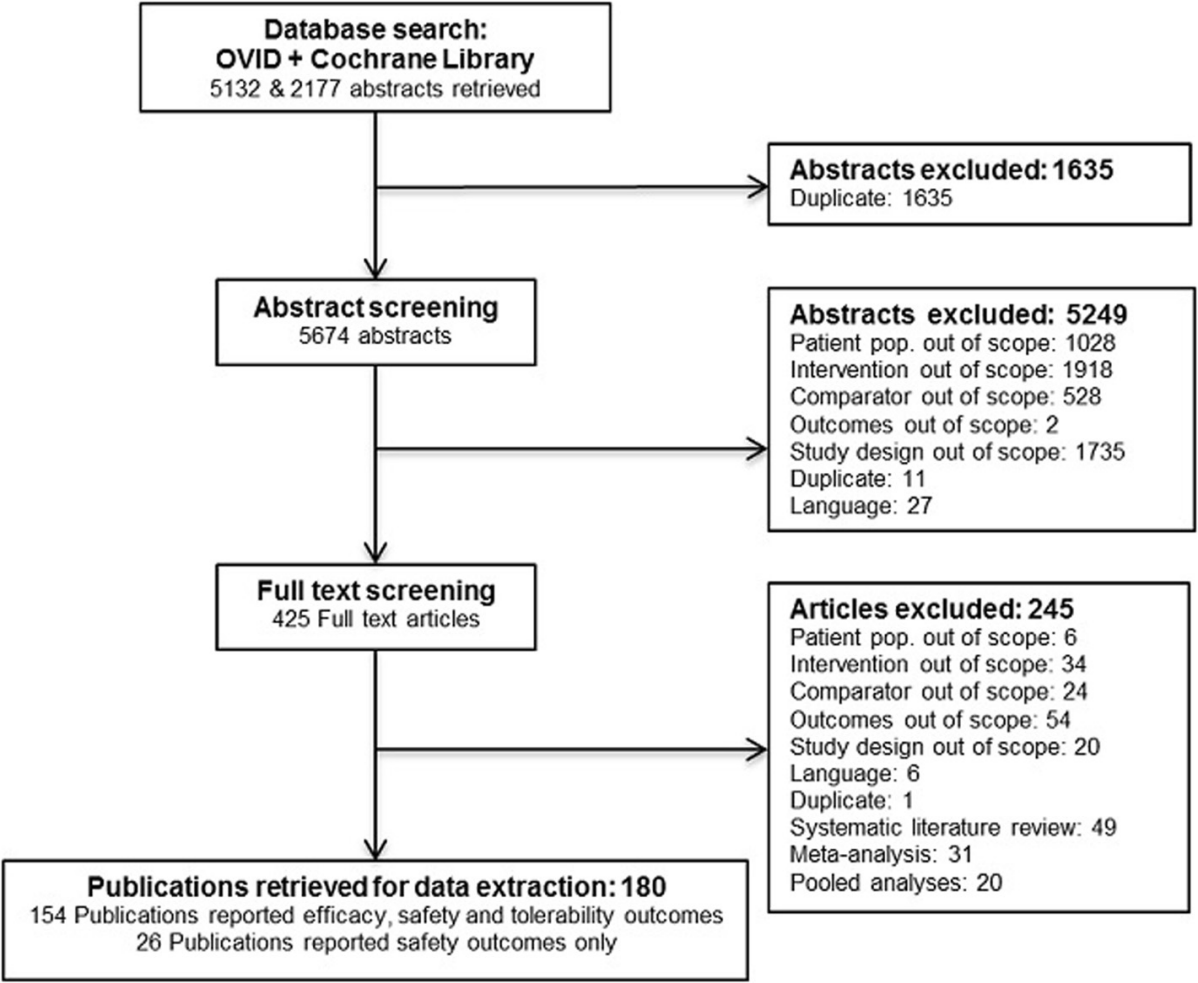
**Study selection flow chart.**

The study selection process is summarised in Figure 2. The database searches performed in June 2013, without restriction on publication year, identified 7,309 citations of which 1,635 were excluded based on duplication elimination. The remaining 5,674 were screened using the population, intervention, comparators, outcomes, and study design (PICOS) criteria and 5,249 were excluded due to interventions not of interest (37%), study design (33%), patient population (20%), and comparators (10%). For the 425 included abstracts, full text publications were retrieved and screened, with 245 being excluded due to outcomes (22%), interventions (14%), comparators (10%), study design (8%), and patient population (2%). A number of pooled analyses (n = 29), systematic literature reviews (SLRs), and (network) meta-analyses of interest (n = 5) were identified and separated from the main base of evidence. Pooled analyses of RCTs were screened for studies not reported elsewhere. One pooled analysis combining three RCTs (not published as independent studies) was identified and added to the evidence base [38]. The SLRs and NMAs were reviewed to validate the results of the selection process, but no further relevant studies were identified [12,13,20,39,40]. Finally, 180 publications, covering 176 individual trials involving 146,524 patients, were identified during the review process and included in the NMA (see Additional file 3 for a complete list of identified studies). Of these, 154 reported relevant efficacy, safety, and tolerability outcomes, while the remaining 26 reported data on safety and/or tolerability outcomes only. Publications covering more than one study were extracted as separate studies, assuming separate randomization schedules were used for each study. Multiple publications covering a single study were grouped together and extracted as one study.

### Study and patient characteristics

The majority of studies included patients with OA (n = 124) and a smaller number of studies investigated an RA population (n = 38) or a combined OA/RA population (n = 14). Most studies reported a randomised (n = 174), double-blind (n = 160), and multicentre study design (n = 128). Two non-randomised studies in which patients served as their own control were included [41,42]. Studies supporting a crossover design, in which patients switched from placebo to active treatment or different dosages of the active substance were included. However, if no washout period between crossover was observed, data on efficacy and safety outcomes after crossover were not used. Overall, the 176 studies included 146,524 patients assigned to one of the interventions of interest, acetaminophen, or placebo. The size of the studies varied, with the number of patients randomised to each treatment ranging from 12 [42] to 6,769 [43]. The trial duration was ranging from 2 to 104 weeks, while most studies lasted 12 weeks (n = 56) or 6 weeks (n = 31). Long follow-up periods were mainly observed in studies investigating the safety of NSAIDs and COXIBs [43-46]. Ninety-five studies were placebo controlled while 80 studies compared active treatments only. Nineteen studies allowed the use of gastro-protective agents during the study, if needed by patients, and 38 studies specifically prohibited their use. Aspirin use was allowed during 66 trials at the discretion of the study investigators, while relevant information was missing for 64 studies. An overview of study design characteristics of included studies is available in Additional file 4.

The age of the enrolled patients ranged from 17 to 75 years. Most studies included a predominantly female population and two included women only [47,48]. Disease duration ranged between 1 and 21 years. Information on underlying risk factors that could act as potential treatment effect modifiers was poorly reported. Eighteen studies reported the percentage of patients with hypertension, mostly recent safety studies with a special interest in CV risk associated with NSAIDs and COXIBs. Fewer studies reported on the percentage of smokers (n = 11), while more information was available on risk factors associated with GI safety, such as history of GI ulcer. Overall, 96 studies excluded patients with a history of GI problems, including active GI ulcer at the screening visit or an ulcer history within 1 to 6 months before enrolment. Details on the patient characteristics of included studies are provided in Additional file 5.

Based on the study design and patient characteristics summarised above, and despite some differences, all 176 studies were considered to be comparable and all studies reporting efficacy and/or tolerability outcomes were included in the analyses. The number of events for safety outcomes in the placebo arms of the studies was limited, with only three APTC events in 630 pyrs of follow-up, four major CV events in 727 pyrs of follow-up, and one major GI event in 548 pyrs of follow-up. Although the incidence of these rare serious events in the placebo arms is expected to be low (especially in studies with relatively short follow-up), this can introduce bias in the analysis [35,36]. For this reason, data were synthesized only if the sum of events per treatment across all included trials in the network was at least 50. As a result, the placebo and acetaminophen arms were not included in the safety analyses networks. Three-arm studies comprising of two active treatment arms and one placebo arm were included in the evidence base, excluding data from the placebo arm. Furthermore, trials with zero events in all arms do not contribute to the evidence on the treatment effect and were thus excluded [32].

### Efficacy and safety outcomes

**Figure 3:**
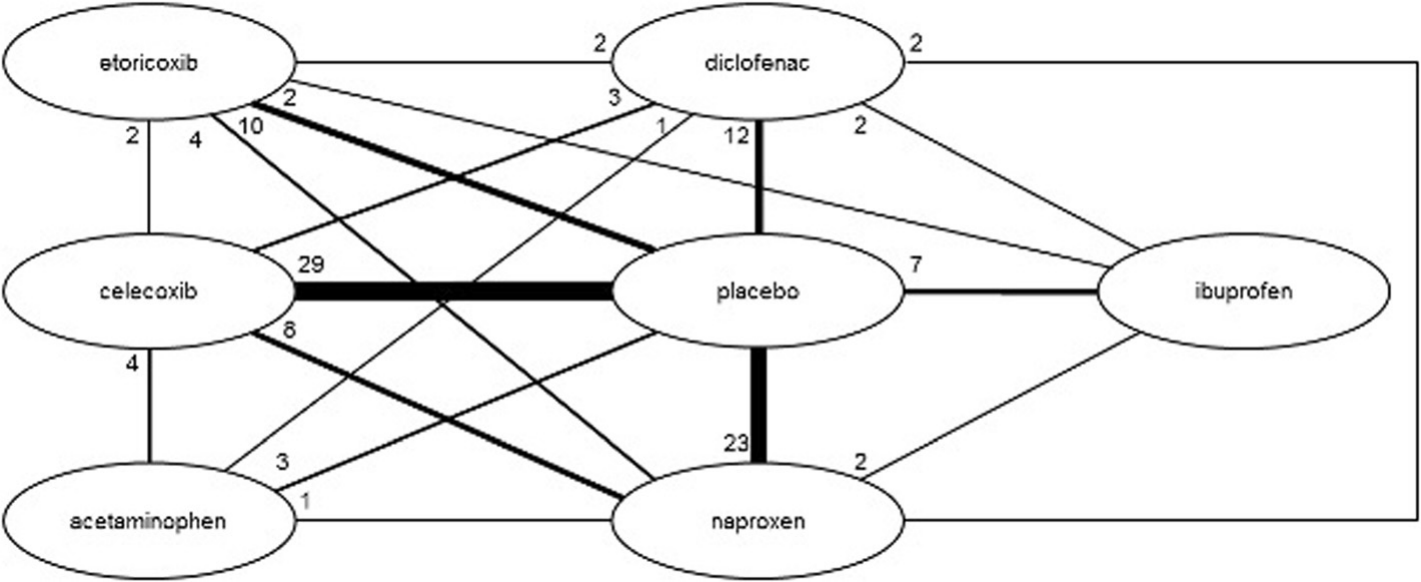
**Evidence network for efficacy outcomes.**

The network diagrams, based on all studies included in the NMA, are presented in Figures 3 (efficacy outcomes) and 4 (safety outcomes). Because not all studies provide data on each outcome, the network diagrams depicting the available evidence per outcome are presented in Additional file 6. The individual study results used for the analyses are presented in Additional file 2.

Efficacy data were synthesized in a base case NMA and seven scenario analyses, listed below. The base case analysis estimated the relative efficacy of the interventions of interest with the maximum dose allowed in OA. Thus, for the base case, diclofenac 150 mg/day was compared to naproxen 1,000 mg/day, ibuprofen 2,400 mg/day, celecoxib 200 mg/day, and etoricoxib 60 mg/day. Next, a number of scenario analyses were performed:

1. Comparative efficacy of a lower diclofenac dose of 100 mg/day, versus the interventions of interest with the maximum dosage allowed in OA
2. Comparative efficacy of 150 mg/day diclofenac versus interventions of interest with the maximum dosage allowed in RA (celecoxib 400 mg/day and etoricoxib 90 mg/day)
3. Comparative efficacy of 100 mg/day diclofenac versus interventions of interest with the maximum dosage allowed in RA (celecoxib 400 mg/day and etoricoxib 90 mg/day)
4. Combining all doses of NSAIDs and COXIBs in OA and RA into a dose range, namely: diclofenac 75 to 150 mg/day, naproxen 500 to 1,000 mg/day, ibuprofen 1,200 to 2,400 mg/day, celecoxib 100 to 400 mg/day, and etoricoxib 30 to 90 mg/day
5. Combining VAS and Likert scales using effect sizes, as described in the Methods section
6. Including studies with at least 100 patients in each treatment arm (data available in Additional file 7)
7. Including only studies recruiting patients after 1999, as a proxy for improved study design according to current standards (data available in Additional file 7).

Majority of safety outcomes (>80%) were reported in trials involving a COXIB or high-dose tNSAID (diclofenac 150 mg/day, ibuprofen 2,400 mg/day, or naproxen 1,000 mg/day). Therefore, for the safety and tolerability outcomes, all available data were pooled and a comparative analysis was conducted.

**Figure 4:**
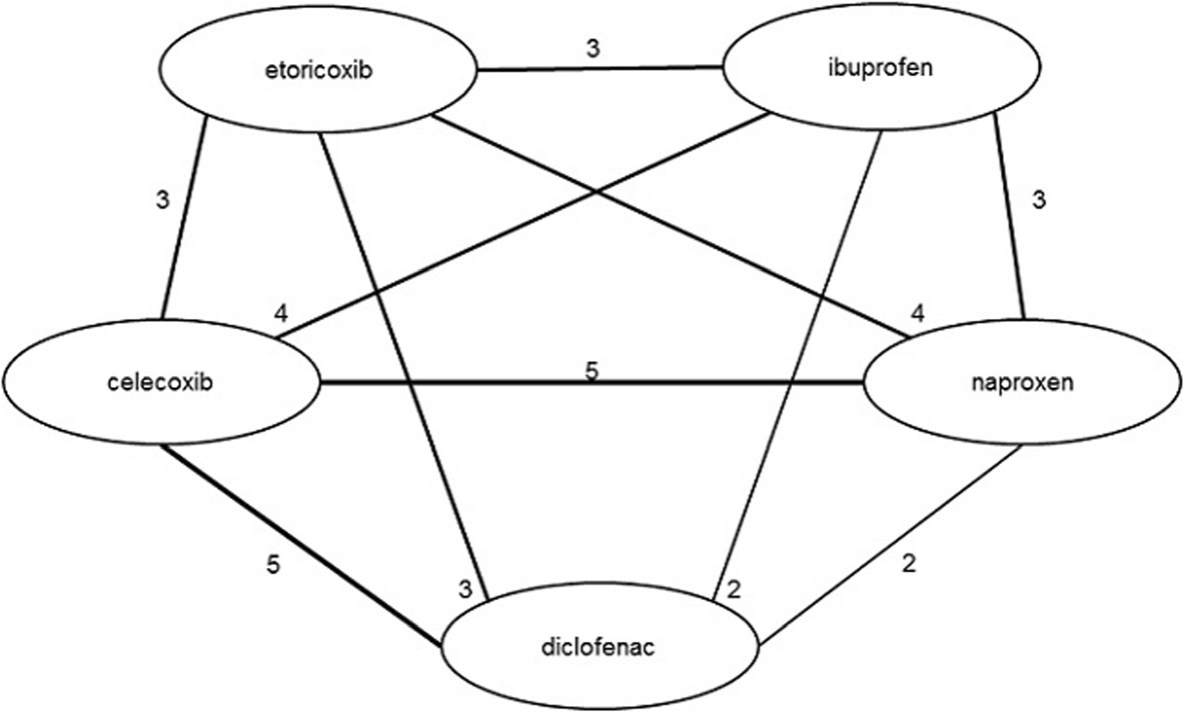
**Evidence network for safety outcomes.**

**Figure 5:**
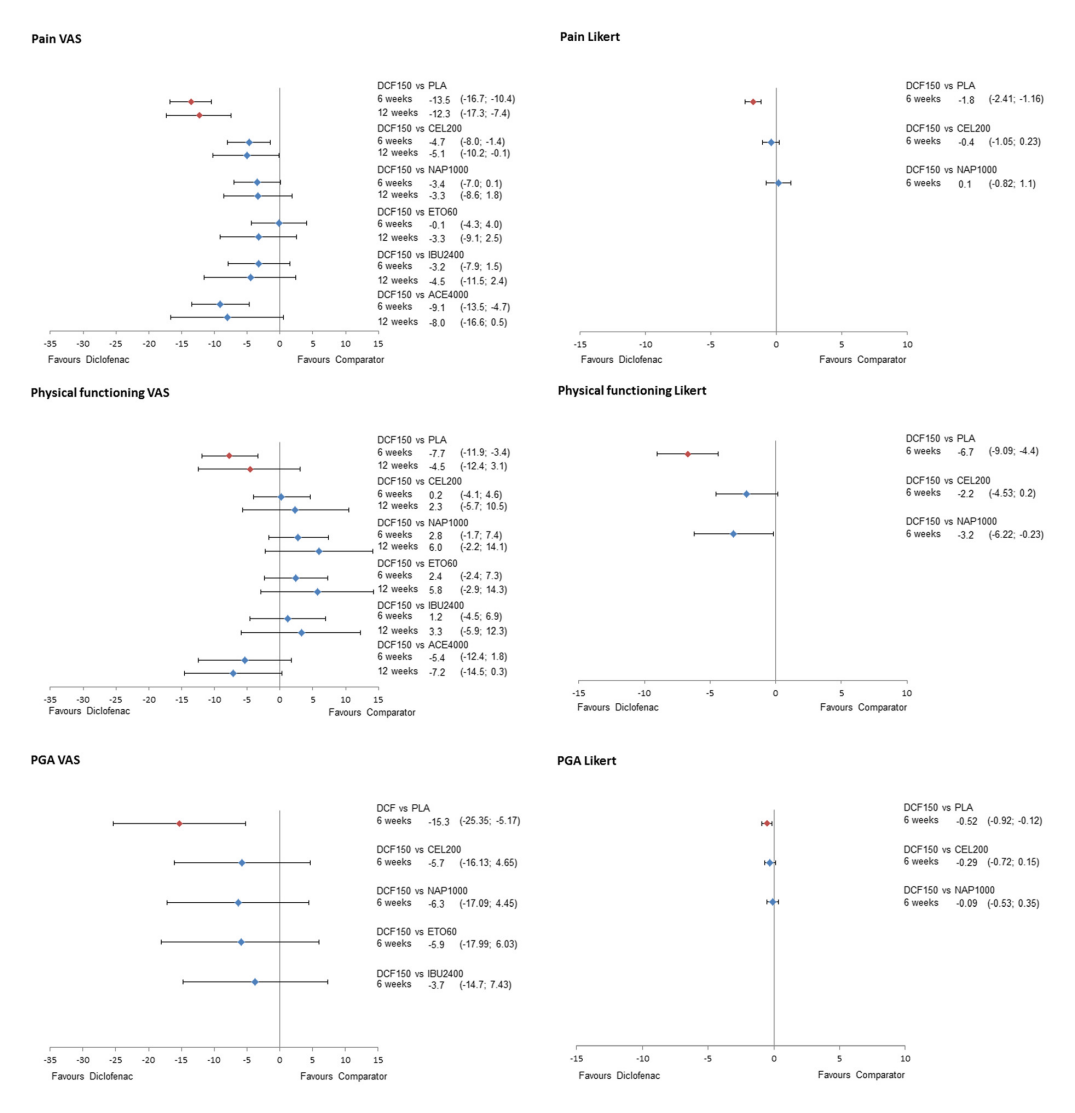
**Forest plots of relative efficacy of diclofenac 150 mg/day.**

**Figure 6:**
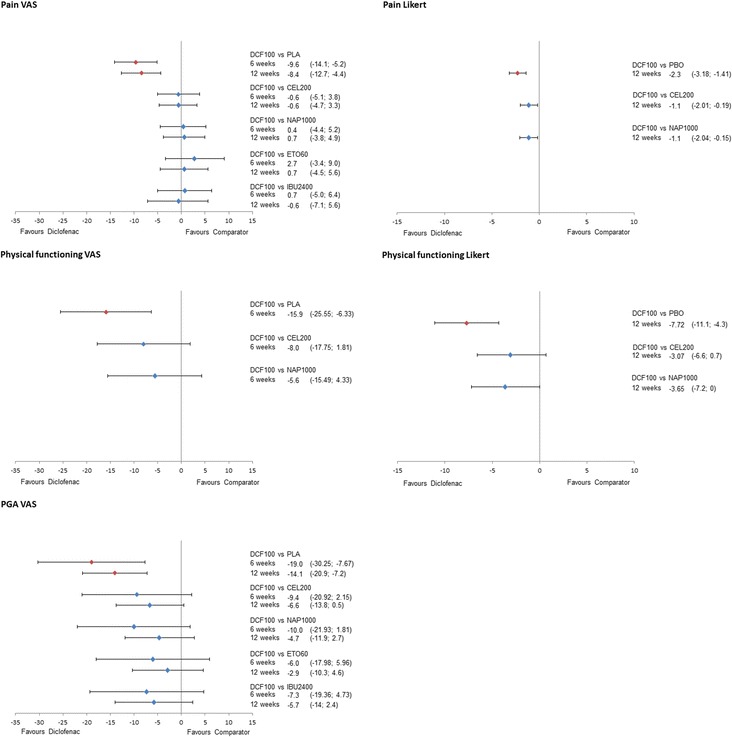
**Forest plots of relative efficacy of diclofenac 100 mg/day.**

The results of the NMA on efficacy outcomes, as ∆CFB with the corresponding 95% CrI for all treatments versus diclofenac are presented in Figures 4 and 5 for the 150 mg/day and 100 mg/day doses of diclofenac, respectively. Results for scenario analyses described above are presented in Additional file 7. The safety and tolerability NMA results, as RRs, are presented in Figure 6 together with their 95% CrI.

### Efficacy

The relative efficacies versus placebo of all included drugs at their maximum recommended doses for OA (diclofenac 150 mg/day, celecoxib 200 mg/day, naproxen 1,000 mg/day, etorixocib 60 mg/day, ibuprofen 2,400 mg/day, and acetaminophen 4,000 mg/day) were evaluated (Table 1). On all efficacy outcomes, all drugs were more efficacious than placebo, with one exception: for physical functioning measured with VAS at 12 weeks, the probability of acetaminophen being better than placebo was only 25%.

**Table 1:** **Relative efficacy versus placebo**

	**Diclofenac 150 mg**		**Celecoxib 200 mg**		**Naproxen 1,000 mg**		**Etoricoxib 60 mg**		**Ibuprofen 2,400 mg**		**Acetaminophen 4,000 mg**	
	**Diff CFB (95% CrI)**	**Prob. better than placebo**	**Diff CFB (95% CrI)**	**Prob. better than placebo**	**Diff CFB (95% CrI)**	**Prob. better than placebo**	**Diff CFB (95% CrI)**	**Prob. better than placebo**	**Diff CFB (95% CrI)**	**Prob. better than placebo**	**Diff CFB (95% CrI)**	**Prob. better than placebo**
Pain VAS 6 weeks	−13.5 (−16.7; −10.4)	>99%	−8.9 (−10.2; −7.6)	>99%	−10.1 (−12.0; −8.2)	>99%	−13.4 (−17.2; −9.7)	>99%	−10.4 (−13.9; −6.8)	>99%	−4.5 (−7.6; −1.3)	>99%
Pain VAS 12 weeks	−12.3 (−17.3; −7.4)	>99%	−7.2 (−8.8; −5.7)	>99%	−9.0 (−10.7; −7.3)	>99%	−9.0 (−12.1; −6.1)	>99%	−7.8 (−12.7; −3.0)	>99%	−4.2 (−12.9; 4.4)	83%
Pain Likert 6 weeks	−1.8 (−2.4; −1.2)	>99%	−1.4 (−1.7; −1.1)	>99%	−1.9 (−2.7; −1.2)	>99%	NA	NA	NA	NA	NA	NA
Pain Likert 12 weeks	NA	NA	−1.2 (−1.5; −1.0)	>99%	−1.2 (−1.6; −0.8)	>99%	NA	NA	NA	NA	NA	NA
PF VAS 6 weeks	−7.7 (−11.9; −3.4)	>99%	−7.9 (−9.8; −6.0)	>99%	−10.5 (−13.0; −8.1)	>99%	−10.2 (−14.4; −5.9)	>99%	−8.9 (−12.7; −5.1)	>99%	−2.4 (−8.4; 3.6)	79%
PF VAS 12 weeks	−4.5 (−12.4; 3.1)	88%	−6.8 (−9.3; −4.9)	>99%	−10.6 (−13.1; −8.0)	>99%	−10.3 (−14.1; −6.6)	>99%	−7.8 (−12.6; −3.2)	>99%	2.5 (−5.0; 10.2)	25%
PF Likert 6 weeks	−6.7 (−9.1; −4.4)	>99%	−4.6 (−5.6; −3.6)	>99%	−3.5 (−5.4; −1.7)	>99%	NA	NA	NA	NA	NA	NA
PF Likert 12 weeks	NA	NA	−4.6 (−5.6; −3.6)	>99%	−4.0 (−5.0; −3.0)	>99%	NA	NA	NA	NA	NA	NA
PGA VAS 6 weeks	−15.3 (−25.4; −5.2)	>99%	−9.6 (−12.0; −7.1)	>99%	−9.0 (−12.7; −5.2)	>99%	−9.4 (−15.9; −2.8)	>99%	−11.6 (−16.2; −7.0)	>99%	NA	NA
PGA VAS 12 weeks	NA	NA	−7.5 (−9.4; −5.7)	>99%	−9.3 (−11.8; −6.8)	>99%	−10.2 (−13.5; −6.9)	>99%	−8.2 (−13.4; −3.0)	>99%	NA	NA
PGA Likert 6 weeks	−0.5 (−0.9; −0.1)	>99%	−0.2 (−0.5; 0.0)	97%	−0.4 (−0.6; −0.2)	>99%	NA	NA	NA	NA	NA	NA
PGA Likert 12 weeks	NA	NA	−0.4 (−0.6; −0.2)	>99%	−0.5 (−0.6; −0.3)	>99%	NA	NA	−0.4 (−0.8; 0.0)	98%	NA	NA

Pain VAS, PF VAS, and PGA VAS are in mm. Pain Likert scale runs from 0 to 20, PF Likert scale runs from 0 to 68, PGA Likert scale runs from 0 to 5. CFB, change from baseline; CrI, credible interval; VAS, visual analogue scale; PF, physical functioning; PGA, patient global assessment; NA, data not available.

The relative efficacy of diclofenac versus the other included drugs on pain, physical functioning, and PGA is described below.

### Pain

Data on pain measured by VAS were reported in 60 studies at 6 weeks and in 36 studies at 12 weeks. No data were available for diclofenac 150 mg/day measured on a Likert scale at 12 weeks and for diclofenac 100 mg/day measured on a Likert scale at 6 weeks.

Diclofenac 150 mg/day demonstrated better results (is likely to be more efficacious) in pain relief on VAS compared to all other treatments in both time points (probability of being better, that is more efficacious, treatment >85% in all pairwise comparisons), with the exception of etoricoxib at 6 weeks (Pr (diclofenac being better) = 52%). At 6 weeks, the ΔCFB compared to celecoxib 200 mg/day was −4.7 (95% CrI −8.0, −1.4), versus naproxen 1,000 mg/day was −3.4 (−7.0, 0), versus etoricoxib 60 mg/day was −0.1 (−4.3, 4.0), and versus ibuprofen 2,400 mg/day was −3.2 (−7.9, 1.5). Differences were similar at 12 weeks, although improved versus etoricoxib (∆CFB −3.3 (−9.1, 2.5)). Favourable results were obtained for pain measured by a Likert scale versus celecoxib (−0.4 (−1.0, 0.2)) and similar versus naproxen (0.1 (−0.8, 1.1)) (Figure 5).

Diclofenac 100 mg/day was comparable to all interventions, both at 6 and 12 weeks on a VAS scale (Figure 6). Pain Likert at 12 weeks showed a favourable result for diclofenac 100 mg/day compared to celecoxib (∆CFB −1.1 (95% CrI −2.0, −0.2)) and naproxen (∆CFB −1.1 (95% CrI −2.0, −0.2)).

### Physical functioning

Physical functioning measured by VAS was reported in 27 studies at 6 weeks and 16 studies at 12 weeks. Fewer studies reported data on a Likert scale; seven studies reported 6-week data and eight studies provided data on 12 weeks.

Diclofenac 150 mg/day showed comparable efficacy on physical functioning (VAS) with celecoxib (6 weeks: ∆CFB 0.2 (−4.1, 4.6); 12 weeks: ∆CFB 2.3 (−5.7, 10.5)) and ibuprofen (6 weeks: ∆CFB 1.2 (−4.5, 6.9); 12 weeks: ∆CFB 3.3 (−5.9, 12.3)), while there was a trend in favour of naproxen and etoricoxib (Figure 5). When physical functioning was measured on a Likert scale at 6 weeks, diclofenac 150 mg/day was likely to be more effective compared to celecoxib (∆CFB −2.2 (−4.5, 0.2)) and more effective than naproxen (∆CFB −3.2 (−6.2, −0.2)). No data were available at 12 weeks.

Data for diclofenac 100 mg/day were only available for physical functioning VAS at 6 weeks and Likert at 12 weeks (Figure 6). In both cases, diclofenac 100 mg/day was likely to be more efficacious (Pr (diclofenac being better) >85%) than the rest of the treatments.

### Patient global assessment

There were 44 studies that provided data on PGA measured by a VAS scale at 6 (24 studies) and 12 (20 studies) weeks, respectively. Data from Likert scale measurements were provided in 14 studies at 6 weeks and 13 studies at 12 weeks. For both diclofenac 150 and 100 mg/day, only one study provided data on PGA VAS at 6 weeks. No data were available for diclofenac 150 mg/day PGA VAS and Likert at 12 weeks and one study reported data for diclofenac 100 mg/day PGA VAS. Likert data were unavailable for diclofenac 100 mg/day at 6 weeks.

When measured on a VAS scale at 6 weeks, the PGA results for diclofenac 150 mg/day are comparable to celecoxib (∆CFB −5.7 (−16.1, 4.7)), naproxen (∆CFB −6.3 (−17.1, 4.4)), etoricoxib (−5.9 (−18.1, 6.0)), and ibuprofen (−3.7 (−14.7, 7.4)) (Figure 5). The results are similar when measured in Likert scale, with diclofenac being comparable to celecoxib (∆CFB −0.3 (−0.7, 0.2)) and naproxen (∆CFB −0.1 (−0.1, 0.4)). No data were available for diclofenac 150 mg/day at 12 weeks.

The results for diclofenac 100 mg/day demonstrated comparable efficacy in terms of PGA VAS at 6 and 12 weeks with etoricoxib 60 mg/day, while it was likely to be more efficacious (Pr (diclofenac being better) >85%) versus all other treatments.

### Scenario analyses

A number of scenario analyses on the efficacy outcomes were performed to test the validity of the results presented above. Comparing diclofenac 150 mg/day or 100 mg/day to the maximum dosages of etoricoxib (90 mg/day) and celecoxib (400 mg/day) allowed in RA did not change the conclusions. Neither did combining all doses into a single dose range for each intervention. Even though a substantial part of the studies identified was relatively old or small, including only recent studies or large studies in the analysis did not influence the relative efficacy of diclofenac compared to other NSAIDs. Lastly, combining different outcome assessment tools, that is VAS and Likert scales, resulted in similar conclusions on efficacy as the base case analysis. Results of all scenario analyses are presented in Table 2 and Additional file 7.

**Table 2:** **Scenario analyses on relative efficacy of diclofenac**

	**Placebo**		**Celecoxib**		**Naproxen**		**Etoricoxib**		**Ibuprofen**		**Acetaminophen**	
**Scenario 4 (dose ranges)**	**Diff CFB (95% CrI)**	**Prob. DCF better**	**Diff CFB (95% CrI)**	**Prob. DCF better**	**Diff CFB (95% CrI)**	**Prob. DCF better**	**Diff CFB (95% CrI)**	**Prob. DCF better**	**Diff CFB (95% CrI)**	**Prob. DCF better**	**Diff CFB (95% CrI)**	**Prob. DCF better**
Pain VAS 6 weeks	−13.7 (−16.9; −10.5)	>99%	−4.9 (−8.3; 1.4)	>99%	−3.1 (−7.1; 0.9)	94%	1.6 (−2.5; 5.6)	23%	−1.3 (−6.7; 4.1)	68%	−9.5 (−14.8; −4.1)	>99%
Pain VAS 12 weeks	−10.1 (−13.2; −7.0)	>99%	−2.6 (−5.8; 0.5)	95%	−0.9 (−4.2; 2.3)	70%	0.4 (−3.4; 4.0)	42%	−1.6 (−5.8; 2.6)	77%	−7.1 (−16.0; 1.7)	94%
Pain Likert 6 weeks	−1.8 (−2.3; −1.2)	>99%	−0.4 (−1.0; 0.2)	90%	−0.3 (−1.1; 0.5)	77%	NA	NA	NA	NA	NA	NA
Pain Likert 12 weeks	−2.3 (−3.4; −1.2)	>99%	−1.2 (−2.3; 0.0)	98%	−1.1 (−2.2; 0.0)	97%	NA	NA	NA	NA	NA	NA
PF VAS 6 weeks	−10.0 (−15.8; −4.4)	>99%	−1.2 (−7.3; 4.6)	66%	0.8 (−5.4; 6.7)	40%	1.2 (−4.4; 6.5)	33%	−1.0 (−7.6; 5.3)	63%	−6.7 (−15.1; 1.2)	95%
PF VAS 12 weeks	−4.5 (−11.9; 2.8)	89%	2.3 (−5.3; 10.0)	27%	5.9 (−1.9; 13.6)	7%	5.3 (−2.4; 13.0)	9%	3.1 (−5.3; 11.4)	23%	−7.1 (−14.2; 0.1)	97%
PF Likert 6 weeks	−6.7 (−9.1; −4.4)	>99%	−2.2 (−4.5; 0.2)	96%	−3.2 (−6.2; −0.2)	98%	NA	NA	NA	NA	NA	NA
PF Likert 12 weeks	−7.7 (−10.7; −4.7)	>99%	−3.8 (−6.9; −0.7)	>99%	−3.6 (−6.7; −0.5)	99%	NA	NA	NA	NA	NA	NA
PGA VAS 6 weeks	−16.6 (−24.3; −9.0)	>99%	−7.1 (−15.1; 0.8)	96%	−7.0 (−15.3; 1.3)	95%	−4.3 (−12.4; 3.8)	85%	−5.3 (−13.9; 3.3)	89%	NA	NA
PGA VAS 12 weeks	−14.1 (−21.4; −6.8)	>99%	−6.6 (−14.1; 0.9)	96%	−4.6 (−12.4; 3.0)	89%	−2.4 (−10.0; 5.2)	74%	−5.5 (−14.1; 3.1)	90%	NA	NA
PGA Likert 6 weeks	−0.5 (−0.7; −0.3)	>99%	−0.2 (−0.4; −0.1)	>99%	−0.1 (−0.2; 0.1)	76%	NA	NA	NA	NA	NA	NA
PGA Likert 12 weeks	NA	NA	NA	NA	NA	NA	NA	NA	NA	NA	NA	NA
**Scenario 5 (effect sizes)**	**Diff CFB (95% CrI)**	**Prob. DCF better**	**Diff CFB (95% CrI)**	**Prob. DCF better**	**Diff CFB (95% CrI)**	**Prob. DCF better**	**Diff CFB (95% CrI)**	**Prob. DCF better**	**Diff CFB (95% CrI)**	**Prob. DCF better**	**Diff CFB (95% CrI)**	**Prob. DCF better**
Pain 6 weeks	−0.7 (−0.9; −0.4)	>99%	−0.3 (−0.5; 0.0)	98%	−0.2 (−0.5; 0.1)	92%	0.0 (−0.3; 0.3)	54%	−0.1 (−0.4; 0.2)	75%	−0.5 (−0.8; −0.1)	>99%
Pain 12 weeks	−0.5 (−0.7; −0.3)	>99%	−0.2 (−0.4; 0.0)	98%	−0.2 (−0.4; 0.0)	95%	−0.1 (−0.3; 0.1)	70%	−0.2 (−0.4; 0.1)	94%	−0.4 (−0.8; 0.1)	93%
PF 6 weeks	−0.5 (−0.6; −0.3)	>99%	−0.1 (−0.3; 0.1)	87%	0.0 (−0.2; 0.2)	51%	0.0 (−0.2; 0.2)	51%	−0.1 (−0.3; 0.1)	83%	−0.3 (−0.5; 0.0)	98%
PF 12 weeks	−0.4 (−0.9; 0.2)	90%	−0.1 (−0.6; 0.5)	60%	0.1 (−0.5; 0.6)	42%	0.0 (−0.5; 0.6)	44%	0.0 (−0.6; 0.5)	55%	−0.6 (−1.1; 0.0)	98%
PGA 6 weeks	−0.8 (−0.9; −0.6)	>99%	−0.2 (−0.4; −0.1)	>99%	−0.3 (−0.5; −0.1)	>99%	−0.2 (−0.4; −0.1)	>99%	−0.4 (−0.6; −0.2)	>99%	NA	NA
PGA 12 weeks	NA	NA	NA	NA	NA	NA	NA	NA	NA	NA	NA	NA

Pain VAS, PF VAS, and PGA VAS are in mm. Pain Likert scale runs from 0 to 20, PF Likert scale runs from 0 to 68, and PGA Likert scale runs from 0 to 5. CFB, change from baseline; CrI, credible interval; VAS, visual analogue scale; PF, physical functioning; PGA, patient global assessment; DCF, diclofenac; NA, data not available.

Scenario 4: combining all doses of NSAIDs and COXIBs in OA and RA into a dose range, namely: diclofenac 75 to 150 mg/day, naproxen 500 to 1,000 mg/day, ibuprofen 1,200 to 2,400 mg/day, celecoxib 100 to 400 mg/day, and etoricoxib 30 to 90 mg/day; Scenario 5: combining VAS and Likert scales using effect sizes, as described in the Methods section.

### Safety

The safety results presented below were based on all available data for the doses (that is, diclofenac 75 to 200 mg/day, naproxen 500 to 1,500 mg/day, ibuprofen 1,200 to 2,400 mg/day, celecoxib 100 to 800 mg/day, or etoricoxib 30 to 90 mg/day).

### APTC

Data on APTC events were reported in 22 studies. Of these, 13 studies were included in the analyses after exclusion of the placebo arms. Most studies reported data for the two COXIBs (seven) and diclofenac (seven). Five studies provided data for ibuprofen and only two studies reported APTC events for naproxen. The longest available follow-up was for diclofenac and etoricoxib (41,225 and 40,578 person years, respectively), with over 96% originating from the MEDAL program. Diclofenac was associated with a similar risk of an APTC event as all other interventions, with an RR of 1.1 (0.7, 1.8) versus celecoxib, 0.9 (0.4, 2.0) versus naproxen, 1.0 (0.9, 1.2) versus etoricoxib, and 0.9 (0.5, 1.6) versus ibuprofen.

### Major CV events

**Figure 7:**
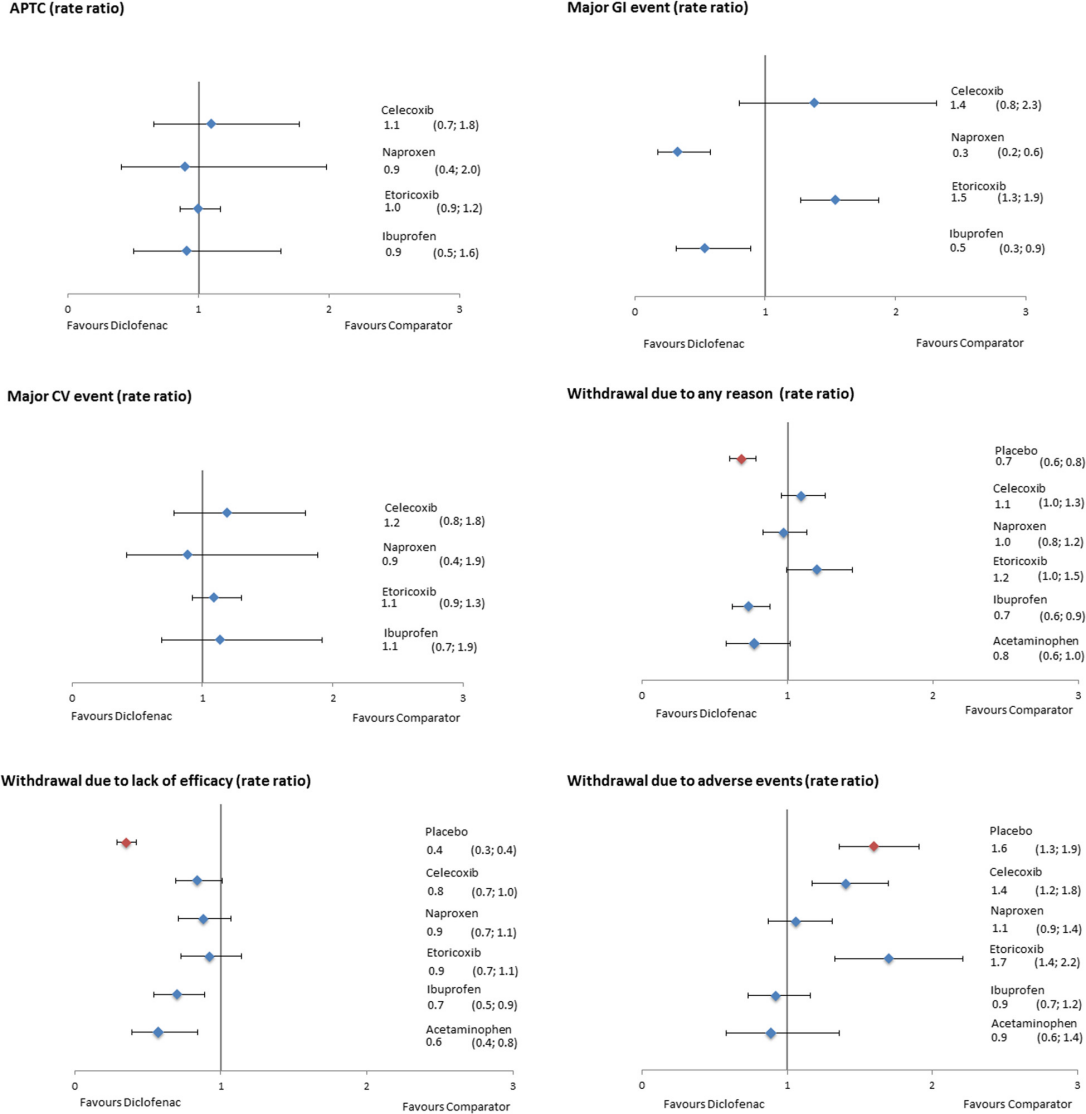
**Forest plots of safety and tolerability outcomes (pooled doses).**

Twenty-six studies provided data on major CV events, of which 15 were included in the NMA. Naturally, all studies reporting APTC events were included in this analysis and two additional studies reporting major CV events for naproxen, etoricoxib, and ibuprofen were identified [49,50]. Etoricoxib studies provided the longest follow-up (26,547 pyrs), with most of the data coming from the MEDAL program (>97% of pyrs). As demonstrated in Figure 7, diclofenac was associated with a similar risk of major CV events as all other interventions, with an RR of 1.2 (0.8, 1.8) versus celecoxib, 0.9 (0.4, 1.9) versus naproxen, 1.1 (0.9, 1.3) versus etoricoxib, and 1.1 (0.7, 1.9) versus ibuprofen. The probability of diclofenac being a safer treatment (that is, reducing the number of events) was low (<25%) for all pairwise comparisons, with the exception of naproxen (62%).

### Major upper GI events

Major upper GI events were reported in 29 studies. The analysis included 20 studies comparing active treatment arms. Most data were available for celecoxib and naproxen, with 10 studies reporting for each treatment. However, the longest follow-up was available for diclofenac (27,300 person years, >90% from MEDAL program). Diclofenac was associated with a lower risk for major upper GI events than both naproxen and ibuprofen, with an RR of 0.3 (0.2, 0.6) versus naproxen and 0.5 (0.3, 0.9) versus ibuprofen. Diclofenac was associated with a comparable risk of major upper GI events compared to celecoxib (RR 1.4 (0.8, 2.3)) and higher compared to etoricoxib (RR 1.5 (1.3, 1.9)).

### Tolerability

The analysis of tolerability outcomes was based on all available data for the dose ranges of interest (diclofenac 75 to 200 mg/day, naproxen 500 to 1,500 mg/day, ibuprofen 1,200 to 2,400 mg/day, celecoxib 100 to 800 mg/day, or etoricoxib 30 to 90 mg/day).

### Withdrawal due to any reason

The number of patients withdrawing for any reason was reported in 96 studies. As demonstrated in Figure 7, diclofenac was associated with a lower risk of withdrawal due to any reason than placebo, ibuprofen, and acetaminophen, with an RR of 0.7 (0.6, 0.8), 0.7 (0.6, 0.9) and 0.8 (0.6, 1.0), respectively. The risk was similar for diclofenac compared to celecoxib and naproxen, with an RR of 1.1 (1.0, 1.3) and 1.0 (0.8, 1.2), respectively. Diclofenac was associated with a higher risk of withdrawal compared to etoricoxib, with an RR of 1.2 (1.0, 1.5) (Figure 7).

### Withdrawal due to adverse events

Patients withdrawing because of adverse events were reported in 105 studies. Diclofenac was comparable to naproxen (RR 1.1 (0.9, 1.4)), ibuprofen (0.9 (0.7, 1.2)), and acetaminophen (0.9 (0.6, 1.4)). The risk was higher for diclofenac compared to placebo (RR 1.6 (1.3, 1.9)), celecoxib (1.4 (1.2, 1.8)), and etoricoxib (1.7 (1.4, 2.2)) (Figure 7).

### Withdrawal due to lack of efficacy

Patients withdrawing because of lack of efficacy were reported in 89 studies. Diclofenac was associated with a lower risk of withdrawals due to lack of efficacy compared to placebo (RR 0.4 (0.3, 0.4)), celecoxib (0.8 (0.7, 1.0)), ibuprofen (0.7 (0.5, 0.9)), and acetaminophen (0.6 (0.4, 0.8)), while the risk was comparable to that of naproxen (0.9 (0.7, 1.1)) and etoricoxib (0.9 (0.7, 1.1)) (Figure 7).

The results of the key benefits and risks for diclofenac 150 mg/day versus the other treatments of interest are summarised together in Table 3.

**Table 3:** **Relative benefits and risks of diclofenac**

	**Outcome**	**Unit**	**Assessment time point**	**Placebo**	**Celecoxib**	**Naproxen**	**Etoricoxib**	**Ibuprofen**	**Acetaminophen**
Benefits	Pain (VAS)	ΔCFB (mm)	6 weeks	−13.5 (−16.7, −10.4)	−4.7 (−8.0, −1.4)	−3.4 (−7.0, 0.1)	−0.1 (−4.3, 4.0)	−3.2 (−7.9, 1.5)	−9.1 (−13.5, −4.7)
		ΔCFB (mm)	12 weeks	−12.3 (−17.3, −7.4)	−5.1 (−10.2, −0.1)	−3.3 (−8.6, 1.8)	−3.3 (−9.1, 2.5)	−4.5 (−11.5, 2.4)	−8.0 (−16.6, 0.5)
	Physical functioning (VAS)	ΔCFB (mm)	6 weeks	−7.7 (−11.9, −3.4)	0.2 (−4.1, 4.6)	2.8 (−1.7, 7.4)	2.4 (−2.4, 7.3)	1.2 (−4.5, 6.9)	−5.4 (−12.4, 1.8)
		ΔCFB (mm)	12 weeks	−4.5 (−12.4, 3.1)	2.3 (−5.7, 10.5)	6.0 (−2.2, 14.1)	5.8 (−2.9, 14.3)	3.3 (−5.9, 12.3)	−7.2 (−14.5, 0.3)
	PGA VAS	ΔCFB (mm)	6 weeks	−15.3 (−25.4, −5.2)	−5.7 (−16.1, 4.7)	−6.3 (−17.1, 4.5)	−5.9 (−18.0, 6.0)	−3.7 (−14.7, 7.4)	NA
Risks	APTC	Rate ratio	Duration of study	NA	1.1 (0.7, 1.8)	0.9 (0.4, 2.0)	1.0 (0.9, 1.2)	0.9 (0.5, 1.6)	NA
	Major CV event	Rate ratio	Duration of study	NA	1.2 (0.8, 1.8)	0.9 (0.4, 1.9)	1.1 (0.9, 1.3)	1.1 (0.7, 1.9)	NA
	Major GI event	Rate ratio	Duration of study	NA	1.4 (0.8, 2.3)	0.3 (0.2, 0.6)	1.5 (1.3, 1.9)	0.5 (0.3, 0.9)	NA
	Withdrawal due to any reason	Rate ratio	Duration of study	0.7 (0.6, 0.8)	1.1 (1.0, 1.3)	1.0 (0.8, 1.2)	1.2 (1.0, 1.5)	0.7 (0.6, 0.9)	0.8 (0.6, 1.0)
	Withdrawal due to adverse events	Rate ratio	Duration of study	1.6 (1.3, 1.9)	1.4 (1.2, 1.8)	1.1 (0.9, 1.4)	1.7 (1.3, 2.2)	0.9 (0.7, 1.2)	0.9 (0.6, 1.4)
	Withdrawal due to lack of efficacy	Rate ratio	Duration of study	0.4 (0.3, 0.4)	0.8 (0.7, 1.0)	0.9 (0.7, 1.1)	0.9 (0.7, 1.1)	0.7 (0.5, 0.9)	0.6 (0.4, 0.8)

Mean and 95% credible intervals are presented; negative ∆CFB and rate ratios <1 favour diclofenac. Benefits were assessed using diclofenac 150 mg/day, naproxen 1,000 mg/day, ibuprofen 2,400 mg/day, celecoxib 200 mg/day, and etoricoxib 60 mg/day. Risks were assessed using dose ranges of the interventions of interest (diclofenac 75 to 200 mg/day, naproxen 500 to 1,500 mg/day, ibuprofen 1,200 to 2,400 mg/day, celecoxib 100 to 800 mg/day, or etoricoxib 30 to 90 mg/day). VAS, visual analogue scale; ΔCFB, difference in change from baseline; PGA, patient global assessment; APTC, Antiplatelets Trialists’ Collaboration; CV, cardiovascular; GI, gastrointestinal.

## Discussion

This study is novel in that a range of different outcomes were brought together, including efficacy (relief of pain, physical functioning, PGA), tolerability (withdrawals), and most commonly studied risks for NSAIDs (GI and CV). Also, the comparison of various benefits and risks was undertaken in a rather homogenous population (arthritis patients) in comparison to previously published meta-analyses of various safety outcomes, which included patients with entirely different underlying conditions, for example Alzheimer’s disease, adenomatous polyps, and others [20,21]. This is in line with the benefit and risk evaluation done for underlying disease conditions in clinical practice and the fact that treatment decisions are dependent upon the defined patient circumstances.

The objective was to assess the efficacy, safety, and tolerability of diclofenac compared to ibuprofen, naproxen, celecoxib, and etoricoxib in patients with pain caused by OA or RA. The analysis was based on RCTs published in peer-reviewed journals. The relevant studies were obtained by means of an SLR and synthesized using a Bayesian NMA, with 176 studies (146,524 patients) providing efficacy, safety, or tolerability data. High-quality SLRs and NMAs were reviewed to validate the results of the selection process, but no further relevant studies were identified.

The efficacy analysis was based on the labelled doses for treatment of OA and RA for each treatment option. All drugs were significantly better than placebo for all efficacy outcomes. Diclofenac 150 mg/day was likely to be more effective in alleviating pain than celecoxib and ibuprofen (both scales: VAS and Likert), naproxen (VAS), and etoricoxib (VAS 12 weeks). Its efficacy was similar compared to etoricoxib (VAS) and naproxen (Likert) at 6 weeks. Diclofenac 100 mg/day was comparable to all other interventions for pain relief. For physical functioning, diclofenac 150 mg/day seemed to be similar to celecoxib and ibuprofen on VAS at 6 and 12 weeks and seemed favourable to celecoxib and naproxen on Likert at 6 weeks. Diclofenac 100 mg/day was comparable to the rest of the treatments for physical functioning VAS at 6 weeks and Likert at 12 weeks, no other data were available. Although only a small number of studies provided data for PGA, diclofenac was comparable to all treatments for the outcomes and time points available. Various scenario analyses were in line with the base case and did not change the main findings.

The safety analysis was based on pooling of events from data available on all doses identified in the evidence base for each treatment because of the low frequency of observed events. All active treatments demonstrated similar incidence of CV outcomes (APTC and major CV). Diclofenac was associated with a lower incidence of major upper GI events compared to naproxen and ibuprofen, comparable to celecoxib, and higher than etoricoxib.

Risk of withdrawals due to any cause was lower for diclofenac than ibuprofen, similar to naproxen and celecoxib, and higher than etoricoxib. Patients treated with diclofenac had a similar risk of withdrawals due to an adverse event to ibuprofen and naproxen and higher risk compared to celecoxib and etoricoxib. The risk of withdrawal due to lack of efficacy for diclofenac was lower than ibuprofen and celecoxib and similar to etoricoxib and naproxen.

The results presented in this study are in agreement with the findings of other Bayesian NMAs of RCTs on the efficacy and safety of COXIBs and NSAIDs published during the last 2 years [20,21,51]. Stam *et al*. compared the efficacy of diclofenac, ibuprofen, naproxen, celecoxib, etoricoxib, and lumiracoxib in OA [51]. Their main findings are in agreement with our results: etoricoxib had a low probability to provide a small improvement in pain relief and physical functioning over diclofenac. For PGA, Stam *et al*. report similar results versus placebo for diclofenac and etoricoxib, while our results are in favour of diclofenac [51]. This could be a result of the differences in the evidence base and methods used (for example all time points were pooled together in Stam *et al*.).

Trelle *at al*. conducted an NMA on the CV safety of the five NSAIDs included in our study plus rofecoxib and lumiracoxib [20]. A much broader patient population (only cancer patients were excluded) and trials with at least 100 pyrs of follow-up per arm were included, leading to a different evidence base compared to our study. Despite this, a similar CV safety profile was reported for diclofenac compared to etoricoxib for all events (RR close to 1), which was confirmed in our findings for major CV and APTC. For celecoxib and ibuprofen, the APTC results versus diclofenac are in agreement with ours, while it is not easy to compare the rest of the outcomes as the mean RR varies per individual CV event. For naproxen, they report either similar results (MI) versus diclofenac or in favour or naproxen (CV death), while in our analysis a mean RR of 0.9 in favour of diclofenac with wide CrIs (0.4, 1.9) were estimated for the major CV events. This difference could be due to the fact that only an inflammatory arthritis population was included in our study.

The CNT Collaboration conducted a meta-analysis of safety outcomes for six COXIBs (celecoxib, etoricoxib, rofecoxib, valdecoxib, lumiracoxib, and GW403681) compared to traditional NSAIDs, including diclofenac [21]. The analysis was based on aggregated and individual patient data from 754 studies, any of which were small and of short duration. Comparing the (pooled) COXIBs to diclofenac, the RR for major vascular events was 0.97 (95% CI 0.84 to 1.12), which is in line with the results of our analysis. Their results are similar for upper GI complications, with an RR of 0.94 (95% CI 0.72 to 1.24) for all COXIBs pooled together; in our study, diclofenac had a comparable profile to celecoxib and etoricoxib demonstrated a better profile. This disagreement could be explained by the difference in the evidence base and the pooling of all COXIBs.

As for any NMA, inherent limitations are related to the quality and availability of data, the potential for within-study bias, and publication bias. Although the studies included were of satisfactory quality, there are limitations to the evidence base, mainly related to the low number of events. A low number of events is limiting the ability of a meta-analysis to detect differences between treatments and can eventually give misleading results [35,36] (also supported by a pain trials simulation study [52]). While we tried to overcome this problem by limiting analyses for safety outcomes to where there were at least 50 events per treatment arm, small differences should still be treated with caution. A publication bias might occur, but we assume that this bias acts in the same way across all the treatments; therefore, in the NMA, the effect of this bias should be ameliorated for the relative difference between comparators. To further reduce the potential of a publication bias, our literature review results were cross-checked with the results of the CNT Collaboration study [21].

Another potential limitation is that studies often use different methods for handling missing data due to dropouts, including last observation carried forward, baseline observation carried forward, multiple imputation, available data, and others. These differences can lead to differences in reported outcomes. However, it has been established that in the absence of a large excess of adverse event withdrawals with active drug over placebo, an imputation method makes little difference [53-56].

Furthermore, a low number of patients or shorter duration can lead to an overestimation of the treatment effect in pain studies and, particularly in OA, over 20% overestimation has been reported by Nuesch *et al.* [57]. Some studies included in this NMA are reporting results for a low number of patients, which may have an impact on the estimation of the outcomes; however, only studies with ≥4 weeks’ treatment duration were included. Various scenario analyses including only studies with more than 100 patients were conducted to validate the results, which were not altered providing additional confidence in the conclusions made.

It could be argued that small changes in pain scales could not be clinically meaningful. However, small average changes on a VAS scale translates into substantial gains in the percentage of people getting good, long-term pain relief. Moore *et al.* reported that an average of 10 mm improvement in pain more than placebo equates to almost one in two patients having substantial benefit [58]. In this analysis, diclofenac 150 mg resulted in an average 12.3 mm improvement over placebo at 12 weeks; therefore, a significant proportion of patients would have had benefit. This was further supported by improvements observed in other benefit outcomes of physical functioning and PGA. Because individual patient data were not available in the publications reviewed, a limitation of our analysis is that it does not report probability of treatments achieving >30% or >50% improvement in pain accepted as clinically meaningful. It is also important to note that general considerations for determining clinically important differences have evolved rather recently [59], and the conduct of most of the clinical studies precede this era. Clinically meaningful differences are not uniformly reported or published. However, clinical response to NSAIDs is highly variable and influenced by a number of factors and is not just limited to the efficacy of individual drugs. No meta-analysis or even a clinical trial is an absolute predictor of meaningful clinical response in the individual patient.

For patients with pain and inflammation, NSAIDs are a recommended treatment option, and it is important to find a balance between known benefits and risks. With good pain relief also come significant improvements in comorbid symptoms like fatigue and depression and large improvements in HRQoL and work [8]. Other treatment alternatives for pain management also have associated risks, for example acetaminophen is associated with liver toxicity and severe cutaneous reactions [60]. Other treatment alternatives include opioids, which could be highly addictive, for example in a cross-sectional study of chronic pain patients, the prevalence of addiction was 14% [61]. Restrictive use of NSAIDs due to the decade-old debate on associated CV risks has led to a drastic increase in opioid prescriptions drug class use, associated with diversion, abuse, overdose, and even deaths due to respiratory depression [62].

NSAIDs have been the cornerstone in pain management for decades, and have a favourable benefit-risk profile and attributes that distinguish them from other available analgesics, also associated with some risks. Patients might be willing to accept risks associated with NSAID treatment for their pain improvement, including a small increase in the risk of serious events, for example in exchange for 25-mm improvement on the VAS scale in ambulatory pain, OA patients were willing to accept an increase of 0.8% points (95% CI 0.4 to 1.4%) in MI risk [63,64]. Risk tolerance is poorly understood and may vary based upon the level of pain, the underlying indication, and the effectiveness and risk associated with a given dose of medication to alleviate painful, debilitating symptoms.

In clinical decision-making, the prescription of treatment involves a trade-off between the expected benefit of treatment and the potential of risk based on patient circumstances. Focusing just on associated risks without taking into consideration the benefits that NSAID treatment can bring could lead to erroneous conclusions in the holistic benefit-risk assessment for these drugs.

This study provides a benefit-risk assessment for a comparative evaluation of the commonly used NSAIDs that could be used to inform clinical practice.

## Conclusions

All NSAIDs were shown to provide clinically meaningful pain relief in patients with chronic arthritis and improve physical function and well-being. The benefit-risk profile of diclofenac was comparable to other treatments used for pain relief in OA and RA. Both benefits and risks vary across treatments and must be taken into consideration while making decisions both by clinicians and by regulators.

## Additional files

Additional file 1:
**Search strategy.**
Additional file 2:
**Input data.**
Additional file 3:
**Included studies.**
Additional file 4:
**Study design tables.**
Additional file 5:
**Patient characteristic tables.**
Additional file 6:
**Scenario analyses.**
Additional file 7:
**Additional scenario analyses.**

## Abbreviations

∆CFB: differences in change from baseline
APTC: Antiplatelet Trialists’ Collaboration
CFB: change from baseline
CNT: Coxib and Traditional NSAID Trialists’
COXIBs: COX 2 inhibitors
CrI: credible interval
CV: cardiovascular
GI: gastrointestinal
HRQoL: Health Related Quality of Life
ITT: intention-to-treat
MCMC: Markov chain Monte Carlo
MI: myocardial infarction
NMA: network meta-analysis
NSAIDS: nonselective nonsteroidal anti-inflammatory drugs
OA: osteoarthritis
PGA: patient global assessment
PICOS: population, intervention, comparators, outcomes, and study design
pyrs: patient years
RA: rheumatoid arthritis
RCTs: randomised controlled trials
RR: rate ratio
SLR: systematic literature review
tNSAIDs: traditional NSAIDs
VAS: visual analogue scale
WOMAC: Western Ontario and McMaster Universities Arthritis Index
