# Supplementary material for: Relative benefit-risk comparing diclofenac to other traditional non-steroidal anti-inflammatory drugs and cyclooxygenase-2 inhibitors in patients with osteoarthritis or rheumatoid arthritis: a network meta-analysis
Source: Arthritis Res Ther. 2015 Mar 19;17(1):66. doi: 10.1186/s13075-015-0554-0 (PMC4411793; doi:10.1186/s13075-015-0554-0)
Supplement: Additional file 1: — Search strategy. [file 13075_2015_554_MOESM1_ESM.docx]

Search strategy for OVID (MEDLINE and EMBASE)

| **ID** | **Search Terms** | **Hits** |  |
| --- | --- | --- | --- |
| 1 | (arthrit$ or osteoarthrit$).mp. | 422748 | Disease terms |
| 2 | exp arthritis/ | 432066 |  |
| 3 | 1 or 2 | 497514 |  |
| 4 | (diclofenac or ibuprofen or naproxen or etoricoxib or arcoxia or mk-663 or mk-0663 or celecoxib or Celebrex or SC-58635).ti,ab,nm. or diclofenac/ or ibuprofen/ or naproxen/ or etoricoxib/ or celecoxib/ | 93372 | Intervention terms |
| 5 | (cyclooxygenase-2 or cyclooxygenase2 or cyclooxygenase-II or cyclooxygenaseII).mp. | 65603 |  |
| 6 | (cyclo oxygenase-2 or cyclo oxygenase2 or cyclo oxygenase-II or cyclo oxygenaseII).mp. | 2772 |  |
| 7 | (cox-2 or cox2 or cox-II or coxII).mp. | 50352 |  |
| 8 | Cyclooxygenase 2 Inhibitors/ | 24477 |  |
| 9 | NSAID$.mp. | 43066 |  |
| 10 | Anti-Inflammatory Agents, Non-Steroidal/ or Analgesics, Anti-Inflammatory/ or Anti-Inflammatory Agents/ | 196998 |  |
| 11 | ((nonsteroid$ or non-steroid$) adj3 (antiinflam$ or antiinflam$)).mp. | 95641 |  |
| 12 | 4 or 5 or 6 or 7 or 8 or 9 or 10 or 11 | 339008 |  |
| 13 | 3 and 12 | 48772 | Study design terms |
| 14 | (Randomized controlled trial or controlled clinical trial).pt. or randomized.ab. or placebo.ab. or clinical trials as topic.sh. or randomly.ab. or trial.ti. or randomised.ti,ab. or exp randomized controlled trial/ | 1656419 |  |
| 15 | 13 and 14 | 8070 |  |
| 16 | (animals not humans).sh. | 3760072 |  |
| 17 | 15 not 16 | 7965 |  |
| 18 | limit 17 to (editorial or letter or note or trade journal or addresses or autobiography or case reports or comment or guideline or interactive tutorial or interview or practice guideline) [Limit not valid in Embase,Ovid MEDLINE(R),Ovid MEDLINE(R) In-Process; records were retained] | 244 |  |
| 19 | 17 not 18 | 7721 |  |
| 20 | Remove duplicates | 6002 |  |
| 21 | Limit to English language | 5132 |  |

Search strategy for Cochrane CENTRAL database

| **ID** | **Search Terms** | **Hits** |  |
| --- | --- | --- | --- |
| 1 | MeSH descriptor: [Arthritis, Rheumatoid] explode all trees | 3982 | Disease terms |
| 2 | MeSH descriptor: [Osteoarthritis] explode all trees | 3097 |  |
| 3 | arthrit* or osteoarthrit* | 12236 |  |
| 4 | #1 or #2 or #3 | 12347 |  |
| 5 | diclofenac or ibuprofen or naproxen or etoricoxib or arcoxia or mk-663 or mk-0663 or celecoxib or Celebrex or SC-58635 | 6611 | Intervention terms |
| 6 | MeSH descriptor: [Diclofenac] explode all trees | 1305 |  |
| 7 | MeSH descriptor: [Ibuprofen] explode all trees | 1114 |  |
| 8 | MeSH descriptor: [Naproxen] explode all trees | 799 |  |
| 9 | MeSH descriptor: [Cyclooxygenase 2 Inhibitors] explode all trees | 485 |  |
| 10 | cyclooxygenase-2 or cyclooxygenase2 or cyclooxygenase-II or cyclooxygenaseII | 945 |  |
| 11 | cyclo oxygenase-2 or cyclo oxygenase2 or cyclo oxygenase-II or cyclo oxygenaseII | 146 |  |
| 12 | cox-2 or cox2 or cox-II or coxII | 844 |  |
| 13 | MeSH descriptor: [Anti-Inflammatory Agents, Non-Steroidal] explode all trees | 6043 |  |
| 14 | (nonsteroid* or non-steroid*) adj3 (antiinflam* or antiinflam*) | 34 |  |
| 15 | #5 or #6 or #7 or #8 or #9 or #10 or #11 or #12 or #13 or #14 | 10719 |  |
| 16 | #4 and #15 | 2301 |  |
| 17 | Limit #16 to Trials | 1961 | Study design terms |
| 18 | Limit #16 from 2003-current AND to Cochrane Reviews (Reviews and Protocols) and Other Reviews | 216 |  |
| 19 | #17 or #18 | 2177 |  |
