# Supplementary material for: Relative benefit-risk comparing diclofenac to other traditional non-steroidal anti-inflammatory drugs and cyclooxygenase-2 inhibitors in patients with osteoarthritis or rheumatoid arthritis: a network meta-analysis
Source: Arthritis Res Ther. 2015 Mar 19;17(1):66. doi: 10.1186/s13075-015-0554-0 (PMC4411793; doi:10.1186/s13075-015-0554-0)
Supplement: Additional file 3: — Included studies. [file 13075_2015_554_MOESM3_ESM.docx]

**Appendix 8. List of included studies**

Accardo S.Seriolo. Controlled-release naproxen in rheumatoid arthritis. Current Therapeutic Research - Clinical and Experimental 1991;49(6):936-42.

Adams MEE. Comparison of Voltaren(TM) SR 75 mg twice daily with Voltaren(TM) 50 mg EC three times daily in the treatment of moderate to severe osteoarthritis of the knee and hip. Current Therapeutic Research - Clinical and Experimental 1990;48(3):476-91.

Altman RDM. Intraarticular sodium hyaluronate (Hyalgan) in the treatment of patients with osteoarthritis of the knee: A randomized clinical trial. Journal of Rheumatology 1998;25(11):2203-12.

Arcangeli P, Andreotti L, Palazzini E, Arcangeli P, Andreotti L, Palazzini E. Effective treatment of osteoarthritis with a 150 mg prolonged-release of diclofenac sodium. Riv Eur Sci Med Farmacol 1996 Sep;18(5-6):217-23.

Arendt-Racine EC, Atkinson MH, Decoteau WE, Flatt VL, Varady J, Arendt-Racine EC, et al. Drug trial in rheumatoid arthritis: a new design. Clinical Pharmacology & Therapeutics 1978 Feb;23(2):233-40.

Baerwald C, V. Efficacy, safety, and effects on blood pressure of naproxcinod 750 mg twice daily compared with placebo and naproxen 500 mg twice daily in patients with osteoarthritis of the hip: A randomized, double-blind, parallel-group, multicenter study. Arthritis Care and Research 2010;62(12):3635-44.

Bakshi R. Comparative efficacy and tolerability of two diclofenac formulations in the treatment of painful osteoarthritis. The British journal of clinical practice 1996;50(6):294-7.

Bakshi RE. Efficacy and tolerability of diclofenac dispersible in painful osteoarthrosis. Clinical rheumatology 1993;12(1):57-61.

Bakshi RE. Efficacy and tolerability of diclofenac dispersible in patients with osteoarthrosis. Current Therapeutic Research - Clinical and Experimental 1992;52(2):307-16.

Bakshi RD. Efficacy and tolerability of diclofenac dispersible in elderly patients with osteoarthritis. Current medical research and opinion 1991;12(7):459-65.

Baraf HSB. Gastrointestinal side effects of etoricoxib in patients with osteoarthritis: Results of the etoricoxib versus diclofenac sodium gastrointestinal tolerability and effectiveness (EDGE) trial. Journal of Rheumatology 2007;34(2):408-20.

Bedogni C, Faini G, Bedogni C, Faini G. Controlled clinical trial on a new nonsteroidal anti-inflammatory drug (NSAID) therapy for arthropathic patients. Riv Eur Sci Med Farmacol 1990 Jun;12(3):197-205.

Bellamy NB. Efficacy and tolerability of enteric-coated naproxen in the treatment of osteoarthritis and rheumatoid arthritis: A double-blind comparison with standard naproxen followed by an open-label trial. Current medical research and opinion 1992;12(10):640-51.

Bensen WW. Efficacy and safety of valdecoxib in treating the signs and symptoms of rheumatoid arthritis: A randomized, controlled comparison with placebo and naproxen. Rheumatology 2002;41(9):1008-16.

Bensen WGF. Treatment of osteoarthritis with celecoxib, a cyclooxygenase-2 inhibitor: A randomized controlled trial. Mayo Clinic Proceedings 1999;74(11):1095-105.

Berry H, Bloom B, Fernandes L, Morris M, Berry H, Bloom B, et al. Comparison of timegadine and naproxen in rheumatoid arthritis. A placebo controlled trial. Clinical rheumatology 1983 Dec;2(4):357-61.

Bhagat K. Effects of non-steroidal anti-inflammatory drugs on hypertension control using angiotensin converting enzyme inhibitors and thiazide diuretics. East African medical journal 2001;78(10):507-9.

Biegert CW, I. Efficacy and safety of willow bark extract in the treatment of osteoarthritis and rheumatoid arthritis: Results of 2 randomized double-blind controlled trials. Journal of Rheumatology 2004;31(11):2121-30.

Bingham III COS. Efficacy and safety of etoricoxib 30 mg and celecoxib 200 mg in the treatment of osteoarthritis in two identically designed, randomized, placebo-controlled, non-inferiority studies. Rheumatology 2007;46(3):496-507.

Birbara CR. Efficacy and safety of rofecoxib 12.5 mg and celecoxib 200 mg in two similarly designed osteoarthritis studies. Current medical research and opinion 2006;22(1):199-210.

Blechman W, Willkens R, Boncaldo GL, Hoffmeister RT, Lockie LM, Multz C, et al. Naproxen in osteoarthrosis. Double-blind crossover trial. Annals of the rheumatic diseases 1978 Feb;37(1):80-4.

Bliddal HR. A randomized, placebo-controlled, cross-over study of ginger extracts and Ibuprofen in osteoarthritis. Osteoarthritis and Cartilage 2000;8(1):9-12.

Bocanegra TSW. Diclofenac/misoprostol compared with diclofenac in the treatment: Of osteoarthritis of the knee or hip: A randomized, placebo controlled trial. Journal of Rheumatology 1998;25(8):1602-11.

Boswell DJO. Evaluation of GW406381 for treatment of osteoarthritis of the knee: Two randomized, controlled studies. MedGenMed Medscape General Medicine 2008;10(11).

Boureau FS. The IPSO study: Ibuprofen, paracetamol study in osteoarthritis. A randomised comparative clinical study comparing the efficacy and safety of ibuprofen and paracetamol analgesic treatment of osteoarthritis of the knee or hip. Annals of the rheumatic diseases 2004;63(9):1028-34.

Bradley JDB. Comparison of an antiinflammatory dose of ibuprofen, an analgesic dose of ibuprofen, and acetaminophen in the treatment of patients with osteoarthritis of the knee. New England Journal of Medicine 1991;325(2):87-91.

Brooks PM, Hill W, Geddes R, Brooks PM, Hill W, Geddes R. Diclofenac and ibuprofen in rheumatoid arthritis and osteoarthritis. Medical Journal of Australia 1980 Jan 12;1(1):29-30.

Buckland-Wright JCM. Quantitative microfocal radiography detects changes in OA knee joint space width in patients in placebo controlled trial of NSAID therapy. Journal of Rheumatology 1995;22(5):937-43.

Caldwell JR, Roth SH, Caldwell JR, Roth SH. A double blind study comparing the efficacy and safety of enteric coated naproxen to naproxen in the management of NSAID intolerant patients with rheumatoid arthritis and osteoarthritis. Naproxen EC Study Group. Journal of Rheumatology 1994 Apr;21(4):689-95.

Caldwell JR, Crain D, Hoffmeister RT, Kantrowitz F, Lloyd RJ, Nussdorf RT, et al. Four-way, multicenter, crossover trial of ibuprofen, fenoprofen calcium, naproxen, and tolmetin sodium in osteoarthritis: comparative clinical profiles. Southern medical journal 1983 Jun;76(6):706-11.

Cannon CPC. A comparison of cardiovascular biomarkers in patients treated for three months with etoricoxib, celecoxib, ibuprofen, and placebo. Archives of Drug Information 2008;1(1):4-13.

Cannon CPC. Cardiovascular outcomes with etoricoxib and diclofenac in patients with osteoarthritis and rheumatoid arthritis in the Multinational Etoricoxib and Diclofenac Arthritis Long-term (MEDAL) programme: a randomised comparison. Lancet 2006;368(9549):1771-81.

Caruso I, Pietrogrande V, Caruso I, Pietrogrande V. Italian double-blind multicenter study comparing S-adenosylmethionine, naproxen, and placebo in the treatment of degenerative joint disease. American Journal of Medicine 1987 Nov 20;83(5A):66-71.

Case JP, Algis J, Balianus JA, Block MD. Lack of efficacy of acetaminophen in treating symptomatic knee osteoarthritis: A randomized, double-blind, placebo-controlled comparison trial with diclofenac sodium. Archives of Internal Medicine 2003;163:169-178.
Castles JJ, Moore TL, Vaughan JH, Bolzan JA, Lee M, Lidsky MD, et al. Multicenter comparison of naproxen and indomethacin in rheumatoid arthritis. Archives of internal medicine 1978 Mar;138(3):362-6.

Cheung R, Cheng TT, Dong Y, Lin HY, Lai K, Lau CS, Feng H, Parsons B. Incidence of gastroduodenal ulcers during treatment with celecoxib or diclofenac: pooled results from three 12-week trials in Chinese patients with osteoarthritis or rheumatoid arthritis. International Journal of Rheumatic Diseases 2010;13:151-157.

Clegg DOR. Glucosamine, chondroitin sulfate, and the two in combination for painful knee osteoarthritis. New England Journal of Medicine 2006;354(8):795-808.

Collantes E.Curtis. A multinational randomized, controlled, clinical trial of etoricoxib inthetreatment of rheumatoid arthritis [ISRCTN25142273]. BMC family practice 2002;3(pp 1-10):1.

Combe BS. Cardiovascular safety and gastrointestinal tolerability of etoricoxib vs diclofenac in a randomized controlled clinical trial (The MEDAL study). Rheumatology 2009;48(4):425-32.

Crook PR, Fowler PD, Hothersall TE, Chiswell RJ, Crook PR, Fowler PD, et al. A study of the efficacy and tolerability of diclofenac and ibuprofen in osteoarthritis of the hip. Br J Clin Pract 1981 Sep;35(9):309-12.

Cryer BLS. A fixed-dose combination of naproxen and esomeprazole magnesium has comparable upper gastrointestinal tolerability to celecoxib in patients with osteoarthritis of the knee: Results from two randomized, parallel-group, placebo-controlled trials. Annals of medicine 2011;43(8):594-605.

Curtis SP, Bockow B, Fisher C, Olaleye J, Compton A, Ko AT, et al. Etoricoxib in the treatment of osteoarthritis over 52-weeks: a double-blind, active-comparator controlled trial [NCT00242489]. BMC musculoskeletal disorders 2005;6:58.

Dahlberg LEH. A randomized, multicentre, double-blind, parallel-group study to assess the adverse event-related discontinuation rate with celecoxib and diclofenac in elderly patients with osteoarthritis. Scandinavian journal of rheumatology 2009;38(2):133-43.

Davies GMW. Comparison of the responsiveness and relative effect size of the Western Ontario and McMaster Universities Osteoarthritis Index and the short-form medical outcomes study survey in a randomized, clinical trial of osteoarthritis patients. Arthritis Care and Research 1999;12(3):172-9.

Day RM. A randomized trial of the efficacy and tolerability of the COX-2 inhibitor rofecoxib vs ibuprofen in patients with osteoarthritis. Archives of internal medicine 2000;160(12):1781-7.

DeLemos BP, X. Tramadol hydrochloride extended-release once-daily in the treatment of osteoarthritis of the knee and/or hip: A double-blind, randomized, dose-ranging trial. American journal of therapeutics 2011;18(3):216-26.

Dieppe PC. A two-year, placebo-controlled trial of nonsteroidal anti-inflammatory therapy in osteoarthritis of the knee joint. British journal of rheumatology 1993;32(7):595-600.

Dreiser RLG. Ibuprofen 800 mg for the treatment of osteoarthritis of the interphalangeal joints of the hand or trapezo metacarpal joint. REV RHUM ENGL ED 1993;60(11):719-24.

Emery PZ. Celecoxib versus diclofenac in long-term management of rheumatoid arthritis: Randomised double-blind comparison. Lancet 1999;354(9196):2106-11.

Emery PK. Analgesic effectiveness of celecoxib and diclofenac in patients with osteoarthritis of the hip requiring joint replacement surgery: a 12-week, multicenter, randomized, double-blind, parallel-group, double-dummy, noninferiority study. Clinical therapeutics 2008;30(1):70-83.

Esselinckx WS. Double-blind comparative study of tenoxicam in arthrosis of the knee and hip. Current Therapeutic Research - Clinical and Experimental 1990;48(1):206-15.

Essex MNB. Efficacy and tolerability of celecoxib versus naproxen in patients with osteoarthritis of the knee: A randomized, double-blind, Double-dummy Trial. Journal of International Medical Research 2012a;40(4):1357-70.

Essex MN, O'Connell M, Bhadra BP, Essex MN, O'Connell M, Bhadra Brown P. Response to nonsteroidal anti-inflammatory drugs in African Americans with osteoarthritis of the knee. Journal of International Medical Research 2012b;40(6):2251-66.

Eversmeyer WP. Safety experience with nabumetone versus diclofenac, naproxen, ibuprofen, and piroxicam in osteoarthritis and rheumatoid arthritis. American Journal of Medicine 1993;95(2 A):2A10S-8S.

Farkouh MEK. Comparison of lumiracoxib with naproxen and ibuprofen in the Therapeutic Arthritis Research and Gastrointestinal Event Trial (TARGET), cardiovascular outcomes: randomised controlled trial. Lancet 2004;364(9435):675-84.

Flavell-Matts SG, Hazleman BL, Houben H, Dhondt E, Tebbs VM. Controlled study of once-daily, sustained release ibuprofen in osteoarthritis. Current Therapeutic Research, Clinical & Experimental 1993;53:394-400.

Fleischmann RMF. A double-masked comparison of Naprelan and nabumetone in osteoarthritis of the knee. Clinical therapeutics 1997;19(4):642-55.

Fleischmann RS. Lumiracoxib is effective in the treatment of osteoarthritis of the knee: A prospective randomized 13-week study versus placebo and celecoxib. Clinical rheumatology 2005;25(1):42-53.

Furst DEK. Dose response and safety study of meloxicam up to 22.5 mg daily in rheumatoid arthritis: A 12 week multicenter, double blind, dose response study versus placebo and diclofenac. Journal of Rheumatology 2002;29(3):436-46.

Gabard BE. Comparative clinical trial with immediate-release diclofenac pellets, 50 mg TID, and slow-release diclofenac pellets, 75 mg BID. Current Therapeutic Research - Clinical and Experimental 1993;54(2):152-60.

Gall EP, Caperton EM, McComb JE, Messner R, Multz CV, O'Hanlan M, et al. Clinical comparison of ibuprofen, fenoprofen calcium, naproxen and tolmetin sodium in rheumatoid arthritis. Journal of Rheumatology 1982 May;9(3):402-7.

Geba GPW. Efficacy of rofecoxib, celecoxib, and acetaminophen in osteoarthritis of the knee: A randomized trial. Journal of the American Medical Association 2002;287(1):64-71.

Germain BF. A placebo-controlled study of diclofenac sodium for the treatment of osteoarthritis of the hip and knee. CURR THER RES, CLIN EXP 1985;37:259-68.

Geusens PA. Efficacy, safety and tolerability of lumiracoxib in patients with rheumatoid arthritis. International journal of clinical practice 2004;58(11):1033-41.

Geusens PPT. A placebo and active comparator-controlled trial of rofecoxib for the treatment of rheumatoid arthritis. Scandinavian journal of rheumatology 2002;31(4):230-8.

Ghosh S.Paul. A study on the effects of diclofenac sodium and etoricoxib in the treatment of osteoarthritis. Journal of the Indian Medical Association 2007;105(5):260-2.

Giansiracusa JE, Donaldson MS, Koonce ML, Lefton TE, Ruoff GE, Brooks CD, et al. Ibuprofen in osteoarthritis. Southern medical journal 1977 Jan;70(1):49-52.

Gibofsky AR. Efficacy and tolerability of valdecoxib in treating the signs and symptoms of severe rheumatoid arthritis: A 12-week, multicenter, randomized, double-blind, placebo-controlled study. Clinical therapeutics 2007;29(6):1071-85.

Gibofsky AW. Comparing the Efficacy of Cyclooxygenase 2-Specific Inhibitors in Treating Osteoarthritis: Appropriate Trial Design Considerations and Results of a Randomized, Placebo-Controlled Trial. Arthritis and rheumatism 2003;48(11):3102-11.

Godfrey RG, de la Cruz S, Godfrey RG, de la Cruz S. Effect of ibuprofen dosage on patient response in rheumatoid arthritis. Arthritis & Rheumatism 1975 Mar;18(2):135-7.

Goldberg MAM. Controlled-release naproxen in the treatment of osteoarthritis. Current Therapeutic Research - Clinical and Experimental 1988;44(1):51-60.

Goldstein JLE. Dyspepsia tolerability from the patients' perspective: A comparison of celecoxib with diclofenac. Alimentary Pharmacology and Therapeutics 2002;16(4):819-27.

Goldstein JLC. Reduced incidence of gastroduodenal ulcers with celecoxib, a novel cyclooxygenase-2 inhibitor, compared to naproxen in patients with arthritis. Am J Gastroenterol 2001;96(4):1019-27.

Goldstein JLC. Celecoxib Plus Aspirin Versus Naproxen and Lansoprazole Plus Aspirin: A Randomized, Double-Blind, Endoscopic Trial. Clinical Gastroenterology and Hepatology 2007;5(10):1167-74.

Gottesdiener KS. Results of a randomized, dose-ranging trial of etoricoxib in patients with osteoarthritis. Rheumatology 2002;41(9):1052-61.

Greenwald MP. Further assessment of the clinically effective dose range of etoricoxib: A randomized, double-blinded, placebo-controlled trial in rheumatoid arthritis. Current medical research and opinion 2011;27(10):2033-42.

Haghighi MK. Comparing the effects of ginger (Zingiber officinale) extract and ibuprofen on patients with osteoarthritis. Archives of Iranian Medicine 2005;8(4):267-71.

Hawel RK. Comparison of the efficacy and tolerability of dexibuprofen and celecoxib in the treatment of osteoarthritis of the hip. International journal of clinical pharmacology and therapeutics 2003;41(4):153-64.

Hawkey CL. Comparison of the effect of rofecoxib (a cyclooxygenase 2 inhibitor), ibuprofen, and placebo on the gastroduodenal mucosa of patients with osteoarthritis: A randomized, double-blind, placebo-controlled trial. Arthritis and rheumatism 2000;43(2):370-7.

Hawkey CCS. Gastroduodenal safety and tolerability of lumiracoxib compared with ibuprofen and celecoxib in patients with osteoarthritis. Journal of Rheumatology 2004;31(9):1804-10.

Hawkey CJL. Incidence of gastroduodenal ulcers in patients with rheumatoid arthritis after 12 weeks of rofecoxib, naproxen, or placebo: A multicentre, randomised, double blind study. Gut 2003;52(6):820-6.

Herrera JAM. Evaluation of the effectiveness and tolerability of controlled-release diclofenac-potassium versus immediate-release diclofenac-potassium in the treatment of knee osteoarthritis. Current Therapeutic Research - Clinical and Experimental 2007;68(2):82-93.

Hochberg MCF. Fixed-dose combination of enteric-coated naproxen and immediate-release esomeprazole has comparable efficacy to celecoxib for knee osteoarthritis: Two randomized trials. Current medical research and opinion 2011;27(6):1243-53.

Humberto-Lizarazo P, Pena CM. Single-blind parallel study comparing naproxen with sulindac and with diclofenac in rheumatoid arthritis. Current Therapeutic Research, Clinical & Experimental 1983;34:701-7.

Hunt RHH. The gastrointestinal safety of the COX-2 selective inhibitor etoricoxib assessed by both endoscopy and analysis of upper gastrointestinal events. Am J Gastroenterol 2003a;98(8):1725-33.

Hunt RHH. Complementary studies of the gastrointestinal safety of the cyclo-oxygenase-2-selective inhibitor etoricoxib. Alimentary Pharmacology and Therapeutics 2003b;17(2):201-10.

Huskisson ECB. Enteric coated naproxen; A double blind trial comparing the tolerance of enteric coated and standard formulations. European journal of rheumatology and inflammation 1992;12(2):27-30.

Huskisson ECS. A clinical comparison of two leading non-steroidal anti-inflammatory drugs. European journal of rheumatology and inflammation 1991;11(2):4-7.

Izhar MA. Effects of COX Inhibition on Blood Pressure and Kidney Function in ACE Inhibitor-Treated Blacks and Hispanics. Hypertension 2004;43(3):573-7.

Katona G, Vargas RB, Katona G, Vargas RB. Double-blind crossover study to evaluate the efficacy of a single daily dose of naproxen in rheumatoid arthritis. European Journal of Rheumatology & Inflammation 1983;6(3):233-41.

Kelly JG, Kinney CD, Devane JG, Mulligan S, Colgan BV, Kelly JG, et al. Pharmacokinetic properties and clinical efficacy of once-daily sustained-release naproxen. European journal of clinical pharmacology 1989;36(4):383-8.

Kirchheiner B, Trang L, Wollheim FA, Kirchheiner B, Trang L, Wollheim FA. Diclophenax sodium (Voltaren) in rheumatoid arthritis: a double-blind comparison with indomethacin and placebo. Int J Clin Pharmacol Biopharm 1976 Jun;13(4):292-7.

Kivitz AE. Randomized placebo-controlled trial comparing efficacy and safety of valdecoxib with naproxen in patients with osteoarthritis. Journal of Family Practice 2002;51(6):530-7.

Kivitz AJN. Reduced incidence of gastroduodenal ulcers associated with lumiracoxib compared with ibuprofen in patients with rheumatoid arthritis. Alimentary Pharmacology and Therapeutics 2004;19(11):1189-98.

Kivitz AJM. Comparative efficacy and safety of celecoxib and naproxen in the treatment of osteoarthritis of the hip. Journal of International Medical Research 2001;29(6):467-79.

Kolodny AL. Two double blind trials of diclofenac sodium with aspirin and with naproxen in the treatment of patients with rheumatoid arthritis. Journal of Rheumatology 1988;15(8):1205-11.

Krueger KL. Gastrointestinal tolerability of etoricoxib in rheumatoid arthritis patients: Results of the etoricoxib vs diclofenac sodium gastrointestinal tolerability and effectiveness trial (EDGE-II). Annals of the rheumatic diseases 2008;67(3):315-22.

The Manchester General Practitioner Group. A study of naproxen and ibuprofen in patients with osteoarthritis seen in general practice. Current Medical Research and Opinion 1984;9(1):41-46.

Laine LM. Ulcer formation with low-dose enteric-coated aspirin and the effect of COX-2 selective inhibition: A double-blind trial. Gastroenterology 2004;127(2):395-402.

Laine LH. A randomized trial comparing the effect of rofecoxib, a cyclooxygenase 2-specific inhibitor, with that of ibuprofen on the gastroduodenal mucosa of patients with osteoarthritis. Gastroenterology 1999;117(4):776-83.

Laine LG. How common is diclofenac-associated liver injury? Analysis of 17,289 arthritis patients in a long-term prospective clinical trial. Am J Gastroenterol 2009;104(2):356-62.

Laine LC. Lower Gastrointestinal Events in a Double-Blind Trial of the Cyclo-Oxygenase-2 Selective Inhibitor Etoricoxib and the Traditional Nonsteroidal Anti-Inflammatory Drug Diclofenac. Gastroenterology 2008;135(5):1517-25.

Laine LC. Assessment of upper gastrointestinal safety of etoricoxib and diclofenac in patients with osteoarthritis and rheumatoid arthritis in the Multinational Etoricoxib and Diclofenac Arthritis Long-term (MEDAL) programme: a randomised comparison. Lancet 2007;369(9560):465-73.

Lanza FLM. Clinical trial: Comparison of ibuprofen-phosphatidylcholine and ibuprofen on the gastrointestinal safety and analgesic efficacy in osteoarthritic patients. Alimentary Pharmacology and Therapeutics 2008;28(4):431-42.

Lehmann RB. Efficacy and tolerability of lumiracoxib 100mg once daily in knee osteoarthritis: A 13-week, randomized, double-blind study vs. placebo and celecoxib. Current medical research and opinion 2005;21(4):517-26.

Lehn OFJ. Enteric-coated and plain naproxen tablets in osteoarthritis; tolerability and efficacy. European journal of rheumatology and inflammation 1992;12(2):31-6.

Leung ATM. Efficacy and tolerability profile of etoricoxib in patients with osteoarthritis: A randomized, double-blind, placebo and active-comparator controlled 12-week efficacy trial. Current medical research and opinion 2002;18(2):49-58.

Lisse JE. Functional status and health-related quality of life of elderly osteoarthritic patients treated with celecoxib. Journals of Gerontology - Series A Biological Sciences and Medical Sciences 2001;56(3):M167-M175.

Lisse JR. Clinical efficacy and safety of Naprelan versus Naprosyn in the treatment of rheumatoid arthritis. American journal of orthopaedics. Belle Mead, N 1996;J. 25(9 Suppl):21-9.

Lister BJP. Efficacy of nabumetone versus diclofenac, naproxen, ibuprofen, and piroxicam in osteoarthritis and rheumatoid arthritis. American Journal of Medicine 1993;95(2A):2A2S-3S.

Lohmander LSM. A randomised, placebo controlled, comparative trial of the gastrointestinal safety and efficacy of AZD3582 versus naproxen in osteoarthritis. Annals of the rheumatic diseases 2005;64(3):449-56.

Makarowski WZ. Efficacy and safety of the COX-2 specific inhibitor valdecoxib in the management of osteoarthritis of the hip: A randomized, double-blind, placebo-controlled comparison with naproxen. Osteoarthritis and Cartilage 2002;10(4):290-6.

March L, I. n Of 1 -trials comparing a non-steroidal anti-inflammatory drug with paracetamol in osteoarthritis. British medical journal 1994;309(6961):1041-6.

Marcolongo R, Giordano N, Bassi GP, Giannini R, Borghi C, Francucci BM, et al. Double-blind preference and compliance multicentre study in osteoarthritis: once-a-day treatment. Clinical rheumatology 1985 Sep;4(3):267-77.

Marcolongo RB. Efficacy of a prolonged-release dosage form of diclofenac sodium in some rheumatic diseases. Current Therapeutic Research - Clinical and Experimental 1996;57(9):711-9.

Matsumoto AKM. A randomized, controlled, clinical trial of etoricoxib in the treatment of rheumatoid arthritis. Journal of Rheumatology 2002;29(8):1623-30.

Mayrhofer F, Mayrhofer F. Efficacy and long-term safety of dexibuprofen [S(+)-ibuprofen]: a short-term efficacy study in patients with osteoarthritis of the hip and a 1-year tolerability study in patients with rheumatic disorders. Clinical rheumatology 2001 Nov;20 Suppl 1:S22-S29.

McKenna FB. Celecoxib versus diclofenac in the management of osteoarthritis of the knee: A placebo-controlled, randomised, double-blind comparison. Scandinavian journal of rheumatology 2001a;30(1):11-8.

McKenna FW. COX-2 specific inhibitors in the management of osteoarthritis of the knee: A placebo-controlled, randomized, double-blind study. Journal of Clinical Rheumatology 2001b;7(3 SUPPL.):151-9.

Mehta S.Dasarathy. A prospective randomized study of the injurious effects of aspirin and naproxen on the gastroduodenal mucosa in patients with rheumatoid arthritis. Am J Gastroenterol 1992;87(8):996-1000.

Meinicke J, Danneskiold-Samsoe B, Meinicke J, Danneskiold-Samsoe B. Diclofenac sodium (Voltaren) and ibuprofen in rheumatoid arthritis. A randomized double-blind study. Scand J Rheumatol Suppl 1980;35:1-8.

Mendelsohn SL. Clinical efficacy and tolerability of naproxen in osteoarthritis patients using twice-daily and once-daily regimens. Clinical therapeutics 1991;13(SUPPL. A):8-15.

Molnar JP, Moxley TE. Ibuprofen (Motrin): a double-blind comparison of two dosages, 2400 mg and 3200 mg daily, for treating rheumatoid arthritis. Current Therapeutic Research Clinical and Experimental 1979;26:581.

Mowat AM, Mowat AG, Jones CW, Engler C, Mowat AM, Mowat AG, et al. A comparison of once and twice daily naproxen in rheumatoid arthritis. Br J Clin Pract 1984 Mar;38(3):95-8.

O'Connor TP, Anderson AM, Lennox B, Muldoon C, O'Connor TP, Anderson AM, et al. A novel sustained-release formulation of ibuprofen provides effective once-daily therapy in the treatment of rheumatoid arthritis and osteoarthritis. Br J Clin Pract 1993 Jan;47(1):10-3.

Pagliara L, Baldelli A, Sanguineti F, Siri R, Pagliara L, Baldelli A, et al. Proxen in the treatment of gonarthrosis. A controlled clinical double-blind study. Drugs Under Experimental & Clinical Research 1986;12(11):929-32.

Pavelka K, Susta A, Kankova D, Vojtisek O, Pavelka K, Susta A, et al. Comparison of three therapeutic regimens using nonsteroidal antirheumatic drugs in patients with rheumatoid arthritis. Zeitschrift fur Rheumatologie 1981 Jul;40(4):165-70.

Petersen SGB. Nonsteroidal anti-inflammatory drug or glucosamine reduced pain and improved muscle strength with resistance training in a randomized controlled trial of knee osteoarthritis patients. Archives of physical medicine and rehabilitation 2011;92(8):1185-93.

Pincus TK. Patient Preference for Placebo, Acetaminophen (paracetamol) or Celecoxib Efficacy Studies (PACES): Two randomised, double blind, placedo controlled, crossover clinical trials in patients with knee or hip osteoarthritis. Annals of the rheumatic diseases 2004;63(8):931-9.

Puopolo AB. A randomized placebo-controlled trial comparing the efficacy of etoricoxib 30 mg and ibuprofen 2400 mg for the treatment of patients with osteoarthritis. Osteoarthritis and Cartilage 2007;15(12):1348-56.

Reginster JYM. Evaluation of the efficacy and safety of etoricoxib compared with naproxen in two, 138-week randomised studies of patients with osteoarthritis. Annals of the rheumatic diseases 2007;66(7):945-51.

Roth SHB. Endoscopic Evaluation of the long term effects of diclofenac sodium and naproxen in elderly patients with arthritis. Clinical drug investigation 1995;9(3):171-9.

Ryley NJC. Clinical evaluation of a new controlled-release formulation of naproxen in osteoarthritis and rheumatoid arthritis. Current medical research and opinion 1988;11(1):16-27.

Saag K, V. Rofecoxib, a new cyclooxygenase 2 inhibitor, shows sustained efficacy, comparable with other nonsteroidal anti-inflammatory drugs: A 6-week and a 1-year trial in patients with osteoarthritis. Archives of family medicine 2000;9(10):1124-34.

Sandelin JH. Local NSAID gel (Eltenac) in the treatment of osteoarthritis of the knee: A double blind study comparing eltenac with oral diclofenac and placebo gel. Scandinavian journal of rheumatology 1997;26(4):287-92.

Sangdee C, Teekachunhatean S, Sananpanich K, Sugandhavesa N, Chiewchantanakit S, Pojchamarnwiputh S, Jayasvasti S. Electroacupuncture versus Diclofenac in symptomatic treatment of Osteoarthritis of the knee: a randomized controlled trial. BMC Complementary and Alternative Medicine 2002;2(3).

Sawitzke ADS. Clinical efficacy and safety of glucosamine, chondroitin sulphate, their combination, celecoxib or placebo taken to treat osteoarthritis of the knee: 2-Year results from GAIT. Annals of the rheumatic diseases 2010;69(8):1459-64.

Scharf Y, Nahir M, Schapira D, Lorber M, Scharf Y, Nahir M, et al. A comparative study of naproxen with diclofenac sodium in osteoarthrosis of the knees. RHEUMATOL REHABIL 1982 Aug;21(3):167-70.

Schiff MH. A comparison of Naprelan and Naprosyn in the treatment of osteoarthritis of the knee. American journal of orthopaedics. Belle Mead, N 1996;J. 25(9 Suppl):14-20.

Schmitt WW. Clinical trial on the efficacy and safety of different diclofenac formulations: Multiple-unit formulations compared to enteric coated tablets in patients with activated osteoarthritis. Inflammopharmacology 1999;7(4):363-75.

Schnitzer TJK. Comparison of the COX-inhibiting nitric oxide donator AZD3582 and rofecoxib in treating the signs and symptoms of osteoarthritis of the knee. Arthritis Care and Research 2005a;53(6):827-37.

Schnitzer TJW. Efficacy of rofecoxib, celecoxib, and acetaminophen in patients with osteoarthritis of the knee. A combined analysis of the VACT studies. Journal of Rheumatology 2005b;32(6):1093-105.

Schnitzer TJF. Lumiracoxib in the treatment of osteoarthritis, rheumatoid arthritis and acute postoperative dental pain: Results of three dose-response studies. Current medical research and opinion 2005c;21(1):151-61.

Schnitzer TJB. Comparison of lumiracoxib with naproxen and ibuprofen in the Therapeutic Arthritis Research and Gastrointestinal Event Trial (TARGET), reduction in ulcer complications: randomised controlled trial. Lancet 2004a;364(9435):665-74.

Schnitzer TJB. Efficacy and safety of four doses of lumiracoxib versus diclofenac in patients with knee or hip primary osteoarthritis: A phase II, four-week, multicenter, randomized, double-blind, placebo controlled trial. Arthritis Care and Research 2004b;51(4):549-57.

Schnitzer TJK. Efficacy and safety of naproxcinod in the treatment of patients with osteoarthritis of the knee: A 13-week prospective, randomized, multicenter study. Osteoarthritis and Cartilage 2010;18(5):629-39.

Schnitzer TJD. A 13-week, multicenter, randomized, double-blind study of lumiracoxib in hip osteoarthritis. Clinical rheumatology 2011;30(11):1433-46.

Schnitzer TJH. Efficacy and Safety of Naproxcinod in Patients with Osteoarthritis of the Knee: A 53-Week Prospective Randomized Multicenter Study. Seminars in arthritis and rheumatism 2011;40(4):285-97.

Sheldon E.Beaulieu. Efficacy and tolerability of lumiracoxib in the treatment of osteoarthritis of the knee: A 13-week, randomized, double-blind comparison with celecoxib and placebo. Clinical therapeutics 2005;27(1):64-77.

Shi W, Wang YM, Li LS, Yan M, Li D, Chen NN, et al. Safety and efficacy of oral nonsteroidal anti-inflammatory drugs in patients with rheumatoid arthritis: a six-month randomised study. Clinical drug investigation 2004;24:89-101.

Sikes DHA. Incidence of gastroduodenal ulcers associated with valdecoxib compared with that of ibuprofen and diclofenac in patients with osteoarthritis. European Journal of Gastroenterology and Hepatology 2002;14(10):1101-11.

Silverstein FEF. Gastrointestinal toxicity with Celecoxib vs nonsteroidal anti-inflammatory drugs for osteoarthritis and rheumatoid arthritis: The CLASS study: A randomized controlled trial. Journal of the American Medical Association 2000;284(10):1247-55.

Simon LSW. Anti-inflammatory and upper gastrointestinal effects of celecoxib in rheumatoid arthritis: A randomized controlled trial. Journal of the American Medical Association 1999;282(20):1921-8.

Singer FM. Evaluation of the efficacy and dose-response relationship of dexibuprofen (S(+)-ibuprofen) in patients with osteoarthritis of the hip and comparison with racemic ibuprofen using the WOMAC osteoarthritis index. International journal of clinical pharmacology and therapeutics 2000;38(1):15-24.

Singh G, Fort JG, Goldstein JL, Levy RA, Hanrahan PS, Bello AE, Andrade-Ortega L, Wallemark C, Agrawal NM, Eisen GM, Stenson WF, Triadafilopoulos G, for the SUCCESS-I Investigators. Celecoxib versus naproxen and diclofenac in osteoarthritis patients: SUCCESS-I study. The American Journal of Medicine 2006;119:255-266.

Smugar SSS. Rofecoxib 12.5 mg, rofecoxib 25 mg, and celecoxib 200 mg in the treatment of symptomatic osteoarthritis: Results of two similarly designed studies. Current medical research and opinion 2006;22(7):1353-67.

Sowers JRW. The effects of cyclooxygenase-2 inhibitors and nonsteroidal anti-inflammatory therapy on 24-hour blood pressure in patients with hypertension, osteoarthritis, and type 2 diabetes mellitus. Archives of internal medicine 2005;165(2):161-8.

Stengaard-Pedersen KE. Celecoxib 200 mg q.d. is efficacious in the management of osteoarthritis of the knee or hip regardless of the time of dosing. Rheumatology 2004;43(5):592-5.

Stock KPG. S-ibuprofen versus ibuprofen-racemate. A randomized double-blind study in patients with rheumatoid arthritis. Rheumatology international 1991;11(4-5):199-202.

Svensson OM. Greater reduction of knee than hip pain in osteoarthritis treated with naproxen, as evaluated by WOMAC and SF-36. Annals of the rheumatic diseases 2006;65(6):781-4.

Tannenbaum HB. Lumiracoxib is effective in the treatment of osteoarthritis of the knee: A 13 week, randomised, double blind study versus placebo and celecoxib. Annals of the rheumatic diseases 2004;63(11):1419-26.

Tannenbaum H, Esdaile J, Topp JR, Rostogi RB. A double-blind, multicenter, controlled study on diclofenac (voltaren TM) and naproxen in patients with rheumatoid arthritis. R.A. CURR THER RES, CLIN EXP 1984;35:357-62.

Temple ARB. Multicenter, randomized, double-blind, active-controlled, parallel-group trial of the long-term (6-12 months) safety of acetaminophen in adult patients with osteoarthritis. Clinical therapeutics 2006;28(2):222-35.

Turner R. Hepatic and renal tolerability of long-term naproxen treatment in patients with rheumatoid arthritis. Seminars in arthritis and rheumatism 1988;17(3 SUPPL. 2):29-35.

Varese C, Palazzini A, Varese C, Palazzini A. Open study of a diclofenac sodium prolonged-release in patients suffering from coxarthrosis. Eur Rev Med Pharmacol Sci 1997 Jan;1(1-3):57-62.

Vetter G, Vetter G. A comparison of naproxen and diclofenac sodium in the treatment of osteoarthritis in elderly patients. Br J Clin Pract 1985 Jul;39(7):276-81.

Wagenitz AM. Comparative efficacy and tolerability of two sustained-release formulations of diclofenac: Results of a double-blind, randomised study in patients with osteoarthritis and a reappraisal of diclofenac's use in this patient population. Current medical research and opinion 2007;23(8):1957-66.

Weisman MH, Weisman MH. Double-blind randomized trial of diclofenac sodium versus placebo in patients with rheumatoid arthritis. Clinical therapeutics 1986;8(4):427-38.

Whelton A, Lefkowith JL, West CR, Verburg KM, Whelton A, Lefkowith JL, et al. Cardiorenal effects of celecoxib as compared with the nonsteroidal anti-inflammatory drugs diclofenac and ibuprofen. Kidney international 2006 Oct;70(8):1495-502.

White WBS. Effects of the Cyclooxygenase Inhibiting Nitric Oxide Donator Naproxcinod Versus Naproxen on Systemic Blood Pressure in Patients With Osteoarthritis. American Journal of Cardiology 2009;104(6):840-5.

White WBF. Comparison of thromboembolic events in patients treated with celecoxib, a cyclooxygenase-2 specific inhibitor, versus ibuprofen or diclofenac. American Journal of Cardiology 2002;89(4):425-30.

Wiesenhutter CWB. Evaluation of the comparative efficacy of etoricoxib and ibuprofen for treatment of patients with osteoarthritis: A randomized, double-blind, placebo-controlled trial. Mayo Clinic Proceedings 2005;80(4):470-9.

Williams HJW. Comparison of naproxen and acetaminophen in a two-year study of treatment of osteoarthritis of the knee. Arthritis and rheumatism 1993;36(9):1196-206.

Williams GWH. Comparison of once-daily and twice-daily administration of celecoxib for the treatment of osteoarthritis of the knee. Clinical therapeutics 2001;23(2):213-27.

Williams GWE. Treatment of osteoarthritis with a once-daily dosing regimen of celecoxib: A randomized, controlled trial. Journal of Clinical Rheumatology 2000;6(2):65-74.

Williams GWK. A comparison of valdecoxib and naproxen in the treatment of rheumatoid arthritis symptoms. Clinical therapeutics 2006;28(2):204-21.

Yocum D, Fleischmann R, Dalgin P, Caldwell J, Hall D, Roszko P, et al. Safety and efficacy of meloxicam in the treatment of osteoarthritis: a 12-week, double-blind, multiple-dose, placebo-controlled trial. The Meloxicam Osteoarthritis Investigators. Archives of internal medicine 2000 Oct 23;160(19):2947-54.

Zacher JF. A comparison of the therapeutic efficacy and tolerability of etoricoxib and diclofenac in patients with osteoarthritis. Current medical research and opinion 2003;19(8):725-36.

Zhao SZF. Evaluation of health-related quality of life of rheumatoid arthritis patients treated with celecoxib. Arthritis Care and Research 2000;13(2):112-21.

Zhao SZM. Evaluation of the functional status aspects of health-related quality of life of patients with osteoarthritis treated with celecoxib. Pharmacotherapy 1999;19(11 I):1269-78.
